# Characterization, Antioxidant Potential, and Pharmacokinetics Properties of Phenolic Compounds from Native Australian Herbs and Fruits

**DOI:** 10.3390/plants12050993

**Published:** 2023-02-21

**Authors:** Akhtar Ali, Jeremy J. Cottrell, Frank R. Dunshea

**Affiliations:** 1 School of Agriculture and Food, Faculty of Sciences, The University of Melbourne, Parkville, VIC 3010, Australia; 2 Faculty of Biological Sciences, The University of Leeds, Leeds LS2 9JT, UK

**Keywords:** medicinal plants, bush tomatoes, bush mint, river mint, sea parsley, antioxidants, LC-MS/MS

## Abstract

In recent decades, plant bioactive phenolic compounds gained much attention due to their various health benefits. Therefore, this study aimed to analyze native Australian river mint (*Mentha australis*), bush mint (*Mentha satureioides*), sea parsley (*Apium prostratum*), and bush tomatoes (*Solanum centrale*) for their bioactive metabolites, antioxidant potential, and pharmacokinetics properties. LC-ESI-QTOF-MS/MS was applied to elucidate these plants’ composition, identification, and quantification of phenolic metabolites. This study tentatively identified 123 phenolic compounds (thirty-five phenolic acids, sixty-seven flavonoids, seven lignans, three stilbenes, and eleven other compounds). Bush mint was identified with the highest total phenolic content (TPC—57.70 ± 4.57 mg GAE/g), while sea parsley contained the lowest total phenolic content (13.44 ± 0.39 mg GAE/g). Moreover, bush mint was also identified with the highest antioxidant potential compared to other herbs. Thirty-seven phenolic metabolites were semi-quantified, including rosmarinic acid, chlorogenic acid, sagerinic acid, quinic acid, and caffeic acid, which were abundant in these selected plants. The most abundant compounds’ pharmacokinetics properties were also predicted. This study will develop further research to identify these plants’ nutraceutical and phytopharmaceutical potential.

## 1. Introduction

The growing interest in phytochemicals for general health to prevent chronic disease and aging fueled nutritionists and other scientists to explore the nature, composition, and presence of bioactive metabolites in plants [1]. It has been demonstrated that some of these bioactive metabolites have curative, preventive, nutritional, and antioxidant properties [2]. Phytochemicals from fruits, vegetables, herbs, spices, and medicinal plants have been extensively studied. Moreover, identifying bioactive metabolites from fruits, herbs, and spices provides the basis for these plants’ putative functionality. In addition to antioxidant functions, phytochemicals play roles in enzyme modulation, cell proliferation and apoptosis, cell transduction, and cell signaling [3].

Plants’ secondary metabolites, particularly polyphenols, have attracted much interest due to their beneficial health properties [4,5]. Australia is enriched with native flora, and possesses around 25,000 species of indigenous plants which have a commercial significance as a novel food source in the medicinal, pharmaceutical, and cosmetic industries due to their rich sources of antioxidant and antimicrobial constituents [6]. Australian herbs and medicinal plants provide novel antioxidant compounds in pharmaceutical, nutraceutical, and functional foods [7]. For decades, herbs and fruits have been used to treat aches, bone fractures, joint inflammation, sprains, and the healing of wounds [8]. Herbs, spices, and fruits are widely used for their health-promoting properties as antidiabetic, antioxidant, anti-inflammatory, antimicrobial, neuro- and cardioprotective, anti-HIV, antipyretic, antihypertensive, and antidepressant agents [9,10,11]. Phenolic metabolites have attracted much interest due to their wide range of proven biological properties. The role of phenolic metabolites in health promotion and disease prevention has been widely studied in recent decades. Phytochemicals, especially polyphenols, have different vital biological activities, including the inhibition of cellular inhibition, signal transduction pathways, enzyme activity, metal chelation, and free radical scavenging capacity in cells [8,11]. Oxidative stress occurs due to the excess of free radicals in the body, while natural antioxidants from native herbs and medicinal plants can inhibit this. Due to chronic oxidative stress, different pathological conditions such as the aging process, cancers, and cardiovascular diseases occur in the human body. Therefore, these native herbs and fruits could be utilized to inhibit the acceleration of these pathological conditions. Bush tomatoes, also known as desert raisin or bush sultana, have been widely grown in the central Australian desert for millennia. The dried bush tomatoes have a piquant and intense caramel flavor, imparting an attractive zest to food products or cuisines. Bush mint, river mint, and sea parsley are other widely used herbs as food flavoring.

Various studies have been conducted to explore the bioactive metabolites [12], but a comprehensive profiling of river mint, bush mint, sea parsley, and bush tomatoes is scarce due to their complex nature, unavailability of commercial standards, and the structure of phytochemicals. The prime purpose of this study was the in-depth profiling of selected plants for the presence of phenolic metabolites important for human health and animal feed. In this perspective, we employed LC-ESI-QTOF-MS/MS to identify and quantify bioactive metabolites from bush mint, river mint, bush tomatoes, and sea parsley. Furthermore, total phenolic content (TPC) and total flavonoid content (TFC) and their antioxidant activities including hydroxyl-radical scavenging activity (^•^OH-RSA), ferric reducing antioxidant power (FRAP), 2,2′-azinobis-(3-ethylbenzothiazoline-6-sulfonic acid (ABTS), 2,2′-diphenyl-1-picrylhy-drazyl (DPPH), ferrous ion chelating assay (FICA), reducing power assay (RPA), and phosphomolybdate assay (PMA) were also quantified. LC-ESI-QTOF/MS-MS is a widely used cutting-edge analytical technique for the profiling of plant extracts due to improved peak resolution, greater authenticity, and high sensitivity. The oral bioavailability, Caco-2 cells, and gastrointestinal absorption, metabolism, distribution, and toxicity of phenolic bioactive metabolites were also evaluated in this study. This study will explore the use of Australian native herbs and fruits in the pharmaceutical, medicinal industry, food, and feed industry due to their potent antioxidant and favorable pharmacokinetics properties.

## 2. Results and Discussion

### 2.1. Estimation of Total Polyphenols and Total Flavonoids

**Figure 1:**
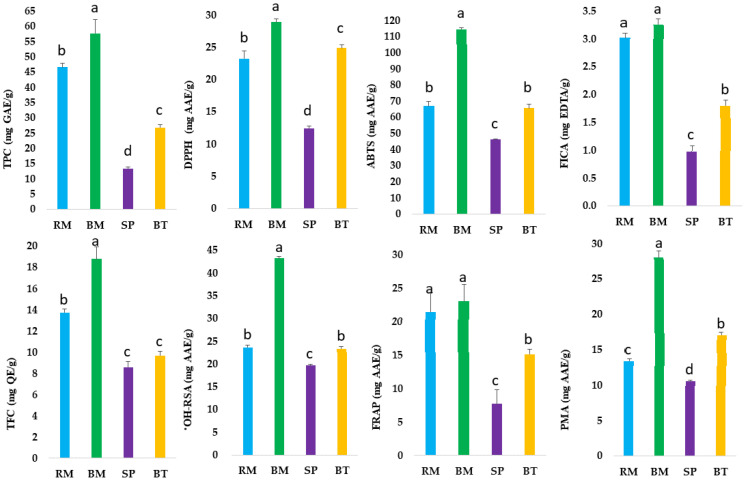
Phenolic contents (TPC and TFC) and antioxidant activities of river mint (RM), bush mint (BM), sea parsley (SP), and bush tomatoes (BT). The vales with letters (a–d) are significantly different from each other.

The interest in improving human and animal health through dietary phytochemicals, especially polyphenols, has increased in recent years [13]. The use of herbs and fruits as sources of bioactive and nutraceutical compounds has attracted much attention from nutritionists and pharmacologists [3]. These bioactive compounds have protective and healing properties. Plant-derived bioactive compounds have various activities in the biological system. Phenolic compounds are the most extensively studied phytochemicals due to their wide range of biological functions and impact on human health [14]. Native Australian herbs and fruits are a rich source of phytochemicals, especially phenolic compounds [15]. In this study, we measured the total phenolic content (TPC) and total flavonoid content (TFC) and their antioxidant activities in river mint, bush mint, sea parsley, and bush tomatoes (Figure 1 and Table S1).

The TPC is usually used to measure the total phenolics including phenolic acids, flavonoids, stilbenes, lignans, and other polyphenols. The highest value of TPC (57.70 ± 4.57 mg GAE/g) was found in bush mint. Overall, native Australian herbs and fruits were observed with an average value of TPC (36.13 mg). The TPC values of bush tomatoes (26.78 ± 1.00 mg GAE/g) and sea parsley (13.44 ± 0.39 mg GAE/g) were also measured. In this study, the level of phenolic contents in bush mint was two- to three-fold higher than lemon myrtle and Tasmanian pepperberry, while the phenolic contents of bush tomatoes and sea parsley were comparable to Australian native Tasmanian pepperberry and lemon myrtle, respectively [15,16]. Furthermore, the phenolic contents in bush mint and river mint were also comparable with Chinese star anise (53.89 ± 1.51 mg GAE/g), citron fruit (46.22 ± 1.01 mg GAE/g), and villous amomum fruit (46.02 ± 1.12 mg GAE/g) [17], while Australian native bush tomatoes, bush mint, and river mint contained higher phenolic compounds than dark plum fruit (11.08 ± 0.19 mg GAE/g), perilla leaf (11.30 ± 0.16 mg GAE/g), peppermint (13.17 ± 0.04 mg GAE/g), black pepper (17.16 ± 0.11 mg GAE/g), and ginger (21.24 ± 0.09 mg GAE/g) [17].

The phenolic contents of native Australian sea parsley (13.44 ± 0.39 mg GAE/g) were comparable to cape jasmine fruit (13.77 ± 0.05 mg GAE/g), kudzu vine root (13.72 ± 0.65 mg GAE/g), and peppermint (13.17 ± 0.04 mg GAE/g), while the phenolic contents of native Australian bush tomatoes (26.78 + 1.00) mg GAE/g) were comparable with mulberry leaf (25.22 + 0.36 mg GAE/g) and Chinse raspberry (23.94 + 0.47 mg GAE/g) [17].

Previously, Sommano et al. [18] measured the total phenolic compounds in bush tomatoes in the range of 7.02 mg/g, while [12] measured the TPC value in the range of 12.4 mg/g which are lower than this study. In our previous study on Australian-grown herbs [3], we measured the total phenolic content (12.43 mg GAE/g/g) in parsley, which is comparatively lower than Australian native sea parsley. The higher value of TPC represents that 80% methanol with 0.1% formic acid allowed better extraction compared to the solvent, time, and other conditions used in the previous study by [12,18]. Other possible reasons might be the different types of cultivars used in current and previous studies. The variation in TPC can also be attributed to various conditions such as solvent, concentration, solvent-to-sample ratio, time and temperature, cultivar, and geographical location where these plants were grown [8]. Furthermore, the methods used to measure the TPC also affect the estimation of phenolics. The TPCs of river mint and bush mint were lower than in a previously conducted study [19].

On the other hand, the TFC values in bush tomatoes (9.66 ± 0.42 mg QE/g) and sea parsley (8.59± 0.51 mg QE/g) were almost the same in both plants’ extracts. The higher TFC value was measured in bush mint (18.81 ± 1.14 mg QE/g) and river mint (13.73 ± 0.32 mg QE/g), respectively. Previously, flavonoids were measured in the range of 8.28–14.7 mg QE/g in river mint, while in this study, river mint was observed 13.73 mg QE/g, which is lower than the previously conducted research [20]. Furthermore, characterization and quantification with LC-MS/MS can deliver more accurate information regarding the presence of individual phenolic metabolites in Australian native herbs and fruits.

### 2.2. Antioxidant Potential of Australian Native Herbs and Medicinal Plants

In this study, a total of six in vitro antioxidant assays were conducted to measure the antioxidant potential of Australian native river mint, bush mint, bush tomatoes, and sea parsley (Table S1, Figure 1).

The DPPH free radical scavenging activity of the selected native Australian plants varied between 12.47 and 28.86 mg AAE/g. Bush mint was quantified with the highest scavenging activity, whereas sea parsley contained the lowest DPPH (12.47 ± 0.35 mg AAE/g) scavenging activity. Previously, Tang et al. [21] quantified the DPPH (110.2 ± 9.0 μmol GAE/g) in native oregano (*Prostanthera rotundifolia*), which is also a type of mint bush. The ABTS assay is an efficient technique to determine the antioxidant activity in plant food extracts as the response of antioxidant ingredients involves rapid reaction kinetics [22]. In this assay, the antioxidant activity of the extracted sample was determined by its reaction with a preformed solution of ABTS^+^ radical cation [3]. The ABTS scavenging activity of the selected native plants was found in the range of 46.18 to 114.44 mg AAE/g (Table S1). The highest ABTS scavenging activity (114.44 ± 1.01 mg AAE/g) was quantified in bush mint, while the lowest ABTS (46.18 ± 0.38 mg AAE/g) activity was quantified in sea parsley. Previously, Tang et al. [21] quantified the ABTS (262.4 ± 2.2 μmol TE/g) in mint bush (*Prostanthera rotundifolia*). The findings of our study are in accordance with the previous studies that reported that herbs and spices with the higher TPC possessed higher antioxidant activity [3,8]. The ABTS scavenging activity was higher than DPPH probably because the ABTS assay was used to measure the antioxidant capacity of hydrophobic and hydrophilic phenolic compounds [23,24]. The ABTS and DPPH of bush mint (114.44 ± 1.01 mg AAE/g) and (28.86 ± 0.49 mg AAE/g) were also found to be higher than what we previously investigated in Australian grown mint (106.99 ± 2.90 mg AAE/g) and (21.65 ± 0.36 mg AAE/g), respectively [3]. This indicates that bush mint has a higher antioxidant potential than mint. The highest FRAP (23.02 ± 2.57 mg AAE/g) activity was quantified in bush mint, while the lowest FRAP (5.13 ± 1.42 mg AAE/g) was quantified in sea parsley.

The metal chelating ability of Australian native herbs and fruits was estimated by using the ferrous ion chelating assay (FICA), and the highest FICA (3.26 ± 0.10 mg EDTA/g) was observed in bush mint. Furthermore, the highest ^•^OH-RSA value (43.25 ± 0.42 mg AAE/g) was also measured in bush mint. This is vital because it inhibits lipid peroxidation by inhibiting the transition of oxidized metal ions [25,26]. It has been reported that there is no single method to measure the total antioxidant potential of plant extracts due to the diverse nature of antioxidant compounds, especially phenolic constituents. The reactive oxygen species (ROS), mainly hydroxyl radical (^•^OH), hydrogen peroxide (H_2_O_2_), and superoxide radical (O_2_), are regularly produced in the human body and harm various cellular biomolecules including protein, carbohydrates, DNA, and lipids leading to different diseases. The highest iron chelating activity observed in bush mint was probably due to the excessive concentration of chelators in its extract. The radical scavenging capacity and reducing ability of iron chelators are dependent on their concentration. A significant variation in the ferrous iron chelating ability of different spices, fruits, vegetables, and root vegetables were also reported [27]. A thorough review of the literature suggests that probably this is the first attempt to determine the scavenging ability of the selected native Australian plants by conducting ^•^OH-RSA and FICA, hence no data are available for comparison. However, in some studies [8], ^•^OH-RSA and FICA assays have been conducted to determine the antioxidant potential of herbs and spices by measuring their potential, and significant variations were recorded in all samples. The variation in results compared to other herbs might be attributed to differences in herb species, maturity stages, agro-ecological conditions, and extraction methods [3,8].

### 2.3. Correlation of Total Phenolic Content and Antioxidant Activities

In this context, we represented the results of phenolic compounds in four Australian native herbs and medicinal plants and their antioxidant potential (Table 1).

A highly significant, positive correlation (*p* ≤ 0.05) of TPC was observed with TFC (*r* = 0.96). The ferric reducing activity of native herbs was strongly correlated with TPC having a Pearson’s correlation coefficient *r* = 0.96 (*p* ≤ 0.05). This positive correlation between TPC and FRAP indicates that the reducing power of the selected native herbs and medicinal plants are strongly linked with their non-flavonoids. These results are in line with findings of Ali et al. [8], who documented that the ferric reducing power ability of dragon fruit was positively associated with its phenolic contents. The ABTS assay established the chain-breaking ability of antioxidants through hydrogen donation by scavenging ABTS^+^ radicals. The strong positive correlation between TPC and ABTS suggests that the selected native Australian herbs contain abundant antioxidants which have a strong ability to scavenge ABTS^+^ radicals by donating hydrogen. The TFC of phenolic extracts of native herbs depicted a highly positive correlation with ^•^OH-RSA (*r* = 0.92, *p* ≤ 0.01) and ferric ion chelating activity (*r* = 0.91, *p* ≤ 0.01). This indicates that the ^•^OH-RSA scavenging activity and ferric ion chelating activity of phenolic extracts of native plants were significantly contributed to by the total flavonoid content. In this experiment, we noted a positive correlation between DPPH and FRAP, FRAP and FICA, and PMA and ^•^OH-RSA. A significant positive correlation between the FRAP and ABTS of spices was also observed previously [3,8].

It has been reported that the DPPH assay is suitable to measure the antioxidant activities of hydrophobic compounds only while the ABTS scavenging assay measures the activity of lipophilic and hydrophilic compounds [3,8,28,29]. This appears to indicate that phenolic compounds in the bush mint samples had a direct relationship with the antioxidant mechanisms of ferric reducing and ferric chelating activity, while flavonoids were more closely associated with the ^•^OH and ferric chelating activities. The results indicate the versality of bioactive compounds in the extracts of native Australian herbs and medicinal plants [30]. Furthermore, the arrangement and number of hydroxyl groups on the ring structure are important to determine the total antioxidant potential of plant extracts [31].

### 2.4. LC-MS/MS Analysis

To confirm the hypothesis that phenolic compounds may contribute to antioxidant activities, phenolic extracts (bush tomatoes, bush mint, river mint, and sea parsley) were further characterized through LC-ESI- QTOF-MS/MS (Figure S1). A total of 123 phenolic metabolites were tentatively identified in these plants (Table 2).

#### 2.4.1. Phenolic Acids

Thirty-five compounds were recognized as phenolic acids. It has been reported that phenolic acids have better sensitivity in the negative mode [32]. Compound 2 (gallic acid—C_7_H_6_O_5_) was identified in bush mint, bush tomatoes, and sea parsley, confirmed through pure standard due to the constant product ion at ESI^−^ *m/z* 125 after the removal of CO_2_ from the precursor ion (*m/z* 169) [8]. Protocatechuic acid 4-*O*-glucoside was identified in sea parsley, bush mint, and bush tomatoes at *m/z* 315.0721 confirmed through the MS/MS product ion at *m/z* 153 after the removal of glycosyl moiety from the precursor ion. Furthermore, protocatechuic acid (compound 5) and p-hydroxybenzoic acid (compound 7) produced fragments at ESI^−^ *m/z* 109 and 93 (Figure S2). Protocatechuic acid is widely distributed in various plants and has antimicrobial, antioxidant, anti-inflammatory, antiviral, anticancer, antiaging, antidiabetic, neuro-protective, cardioprotective, and hepatoprotective properties [33].

Rosmarinic acid, chicoric acid, ferulic acid, sinapic acid, 3-caffeoylquinic acid (chlorogenic acid), caffeic acid, *p*-coumaric acid, syringic acid, and cinnamic acid were confirmed through pure standards. Compound 22 (*p*-coumaric acid—C_9_H_8_O_3_) was putatively identified in bush tomatoes, bush mint, river mint, and sea parsley in the negative mode at *m/z* 119 after the loss of CO_2_ [M−H−44]^−^ from the precursor ion (*m/z* 163.0395) (Figure S3). Ferulic acid (compound 23) was confirmed through the pure standard at *m/z* 193.0504 in river mint and sea parsley. Ferulic acid has been reported for a wide range of therapeutic properties including antioxidant, anticancer, antidiabetic, antiapoptotic effect, antiaging effect, neuro-protective effect, radioprotective effect, pulmonary protective effect, hypotensive effect, and antiatherogenic effect [34]. Furthermore, 3-feruloyqunic acid (compound 24) was detected in river mint and bush tomatoes, while 1,5-dicaffeoylquinic acid (compound 29) was detected in sea parsley, bush mint, and bush tomatoes in both modes. Compound 30 (*m/z* 179.0353) was identified as caffeic acid after producing a product ion at ESI^−^ *m/z* 135 (Figure S2). Rosmarinic acid produced a characteristic fragment at *m/z* 197, which was a 2-hydroxy derivative of hydrocaffeic acid, while two caffeic acid fragments at 161 and 135 represented the removal of H_2_O and CO_2_ (Figure S2). Rosmarinic acid (compound 33) was one of the most abundant phenolic acids commonly present in these herbs. It has various health benefiting properties such as anti-inflammatory, antidepressant, antiulcerogenic, antioxidant, and antimicrobial properties which were widely studied in different studies [35,36]. Previously, it was also identified and quantified in oregano, rosemary, mint, basil, bay, and thyme [3].

#### 2.4.2. Flavonoids

Sixty-seven flavonoids were detected in the selected plants. Compound 37 (*m/z* 289.0704) was detected in bush mint and bush tomatoes in the negative mode. Compound 37 was tentatively identified as epicatechin (C_15_H_14_O_6_) [37]. Procyanidin dimmer B2 (compound 36) was detected in bush tomatoes which made product ions at *m/z* 451, 525, 407, and *m/z* 289. Procyanidin trimmer C1 (compound 38) was found in bush mint at ESI^−^ *m/z* 865.2004. Previously, procyanidin dimmer B1 and procyanidin trimmer C1 were detected in nutmeg and cinnamon [8]. Compound 44 (neoeriocitrin) was detected in bush tomatoes, bush mint, and river mint. Previously, Zeng et al. [38] also reported neoeriocitrin in the extract of Exocarpium Citri grandis (ECG). Naringin (compound 47) at ESI^−^ was putatively identified in bush tomatoes, bush mint, river mint, and sea parsley, which produced daughter ions at *m/z* 459, *m/z* 313, and *m/z* 271 after the loss of [M–H−C_8_H_8_O]^−^, [M–H–C_8_H_8_O-rha]^−^, and [M–H–rha-glu]^−^ from the precursor ion. Compound 58 at ESI^−^ *m/z* 285.0403 produced fragment ions at m/z 177, 151, and 119 after the loss of C_6_H_6_O [M-H-94], C_8_H_8_O [M-H-120], and C_7_H_7_O_4_ [M-H-152], respectively, from the precursor ion. Compound 58 was tentatively identified as 3,4′,7-Tetrahydroxyflavone. Compounds 54 (kaempferol), 57 (swertisin), 60 (diosmin), 63 (diosmetin), and 66 (chrysin) were identified through the MS/MS spectra of pure standards.

Kaempferol 3,7-*O*-diglucoside (compound 77) at ESI^−^ *m/z* 609.1459 was tentatively identified in bush tomatoes, bush mint, river mint, and sea parsley, which generated product ions at *m/z* 447 and *m/z* 285 after the loss of [M−H−162]^−^ and [M−H−324]^−^, respectively, detected in MS/MS. Myricetin 3-*O*-rhamnoside (compound 85), myricetin 3-*O*-rutinoside (compound 78), myricetin 3-*O*-arabinoside (compound 83), isorhamnetin 3-*O*-rutinoside (compound 74), isorhamnetin 3-*O*-glucuronide (compound 80), quercetin 4′-*O*-glucuronide (compound 82), kaempferol 3-*O*-glucuronide (compound 79), and jaceidin 4′-*O*-glucuronide (compound 81) produced fragment ions at *m/z* 317 (myricetin), *m/z* 315 (isorhamnetin), *m/z* 301 (quercetin), *m/z* 285 (kaempferol), and *m/z* 359 (jaceidin) after the loss of rhamnoside [M−H−146]^−^, rutinoside [M−H−308]^−^, arabinoside [M−H−132]^−^, and glucuronide [M−H−176]^−^ from their precursor ions, respectively (Table 2). Previously, myricetin 3-*O*-rhamnoside and quercetin 4′-*O*-glucuronide were reported in lemon and mint with a strong antioxidant potential [39]. Compound 86 (3,7-dimethylquercetin) was detected in bush tomatoes, bush mint, and river mint [40]. Previously, it had been identified in mint, rosemary, sage, basil, and oregano [3]. Compound 77 (kaempferol 3,7-*O*-diglucoside) at ESI^−^ *m/z* 609.1459 produced fragment ions at *m/z* 447 and *m/z* 287 after the loss of one glucoside [M−H−162] and two glucoside units [M−H−324].

#### 2.4.3. Stilbenes and Lignans

Stilbenes and lignans are vital phenolic compounds due to their potent health effects. In this experiment, we putatively identified nine phenolic metabolites in selected herbs and medicinal plants. Piceatannol (compound 103) at ESI^−^ *m/z* 243.0643 generated a product ion at *m/z* 225 after the loss of H_2_O (18) from the precursor ion. Piceatannol was identified in sea parsley, river mint, and bush tomatoes. It has some well-known health properties such as antioxidant, antimutagenic, anticancer, and anti-inflammatory elements; see Ali et al. [3]. Compound 109 (*m/z* 361.1661) produced a product ion at *m/z* 346 after the removal of methyl radical from the precursor ion, while the same compound also produced product ions at *m/z* 177 and 165 after the C8-C8′-carbons’ cleavage from the parent ion. Previously, Hanhineva et al. [41] also defined the presence of secoisolariciresinol through MS/MS detected in whole-grain rye bran. Compound 106 (sagerinic acid) was only identified in bush mint. Previously, Velamuri et al. [42] identified and quantified sagerinic acid in sage and rosemary, while Serrano et al. [43] also reported sagerinic acid in *Lepechinia meyenii* (Walp.) Epling and *Lepechina foribunda* (Benth.) Epling. Lu and Yeap Foo [44] also reported the antioxidant activity of sagerinic acid. Sagerinic acid is widely distributed in herbs and spices. Compound 112 (enterolactone) at ESI^+^ *m/z* 299.1279 was tentatively identified in bush mint, river mint, and sea parsley and has been reported for having antioxidant [45] and anticancer activities [46].

#### 2.4.4. Other Compounds

Phenolic terpenes including carnosol and carnosic acid were reported by Wang et al. [47]. Compound 121 and 122 (carnosol and carnosic acid) generated fragment ions at *m/z* 285 and *m/z* 287 via the removal of CO_2_ (44) from their precursor ions, respectively. The antioxidant potential of phenolic terpenes was reported by Zabot et al. [48] for the prevention of various pathologies in the pharmaceutical area. Both phenolic terpenes were reported in bush mint and river mint. Compound 113 (scopoletin) at ESI^−^ *m/z* 191.0355 was identified in bush mint, sea parsley, and bush tomatoes which produced a fragment ion at *m/z* 147 after the loss of CO_2_ from the precursor ion. Compound 117 (umbelliferone) was in all selected native herbs, while coumarin (compound 119) was detected through MS/MS product ions in bush mint, river mint, and bush tomatoes. Previously, coumarin was identified in cinnamon, fennel, allspice, and oregano, while umbelliferone was identified in mint, rosemary, oregano, sage, and basil [3,8].

### 2.5. Distribution of Bioactive Phenolic Metabolites in Selected Native Australian Plants

**Figure 2:**
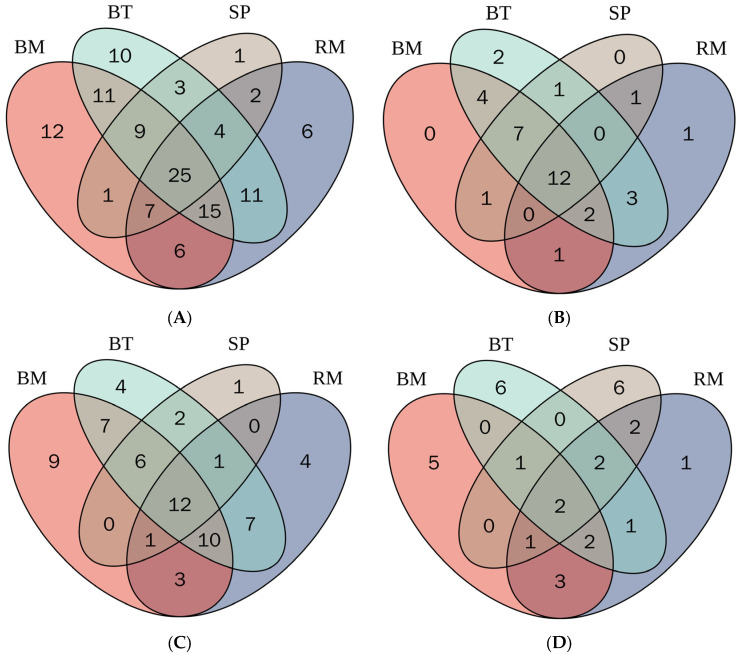
Distribution of phenolic compounds in native Australian river mint (RM), bush mint (BM), bush tomatoes (BT), and sea parsley (SP). (**A**) Total phenolic compounds; (**B**) total phenolic acids; (**C**) total flavonoids; (**D**) total other compounds in native Australian plants.

The distribution of phenolic compounds in the selected native Australian plants was achieved by conducting the Venn diagram represented in Figure 2.

Native Australian herbs and fruits contain a diverse range of different phenolic compounds including total phenolics, total phenolic acids, total flavonoids, and total other polyphenols including stilbenes, lignans, phenolic terpenes, curcuminoids, and tyrosols, etc.

The Venn diagram (Figure 2A) represents that the highest total number of unique metabolites was identified in bush mint (12, 9.8%), while the total lowest number of unique metabolites was identified in sea parsley (1, 0.8%). In addition, 25 metabolites (20.0%) were overlapped in river mint, bush mint, bush tomatoes, and sea parsley. The results clearly demonstrate that bush mint contained a wide range of phenolic metabolites that contributed to the higher antioxidant potential. Moreover, the distribution of the total number of phenolic acids is represented in Figure 2B, which depicts that the highest number of unique phenolic acids was identified in bush tomatoes (2, 5.7%). Twelve phenolic acids were overlapped in bush mint, river mint, bush tomatoes, and sea parsley. Total flavonoids are represented in Figure 2C, which shows that the highest number of total unique flavonoids was detected in bush mint (9, 13.0%), while the lowest number of unique total flavonoids was detected in sea parsley (1, 1.5%). In flavonoids, twelve metabolites (18.0%) were present in all herbs and fruits, while seven (10.0%) compounds were overlapped in bush mint and bush tomatoes. Figure 2D was conducted to represent the distribution of the total other compounds (stilbenes, lignans, phenolic terpenes, curcuminoids, and tyrosols) in river mint, bush mint, bush tomatoes, and sea parsley. It depicts that the highest number of unique other metabolites (6, 19.0%) was identified in bush tomatoes and sea parsley, while the lowest number of other phenolic metabolites (1, 3.1%) was in river mint. Two (6.2%) other phenolic metabolites were overlapped in river mint, bush tomatoes, and sea parsley, while two (6.2%) other compounds were overlapped in river mint and sea parsley.

### 2.6. LC-MS/MS Quantification/Semi-Quantification of Individual Phenolic Metabolites

Natural products have been used to improve human health for years. The nutraceutical usage of fruits as protective and healing supplements has been increased due to their wide range of bioactive phenolic and non-phenolic metabolites. Polyphenols are organic compounds which are derived from plant-based foods, and they play a significant role in the prevention of many oxidative stress-related diseases such as cardiovascular, cancers and neurodegenerative diseases.

#### 2.6.1. Phenolic Acids

A total of 17 phenolic acids were semi-quantified in Australian native herbs and fruits (Table S2). Rosmarinic acid was the most abundant phenolic metabolite found in bush mint (945.56 ± 43.50 μg/g) and river mint (745.67 ± 25.02 μg/g). It was also quantified in bush tomatoes (76.57 ± 4.98 μg/g) and sea parsley (23.43 ± 1.01 μg/g). The higher concentration of chlorogenic acid was quantified in bush tomatoes (747.52 ± 67.48 μg/g), bush mint (584.07 ± 12.39 μg/g), river mint (238.76 ± 14.99 μg/g), and sea parsley (29.07 ± 0.98 μg/g), respectively. Previously, rosmarinic acid was also quantified in oregano (1.6 mg/g), rosemary (0.54 mg/g), mint (0.20 mg), and other herbs [3]. Tang et al. [19] also quantified chlorogenic acid in Australian native mint (15.4 μg/mg of purified extract). Rosmarinic acid is the biomarker of herbs; therefore, it is widely identified and quantified in herbs. Previously, Tang et al. [19] also quantified rosmarinic acid in native mint (160.4 μg/mg of purified extract). Moreover, Wang et al. [49] quantified rosmarinic acid in the range of 2.0–27.4 mg/g, while Zheng and Wang [50] quantified rosmarinic acid in the range of 0.33–1.5 mg/g in different Mediterranean herbs through HPLC. We found only one study where they observed the antioxidant activities of Australian native bush mint, and only eight phenolic compounds were identified and quantified [21]. Both gallic acid and vanillic acid were quantified in bush tomatoes, bush mint, and sea parsley. Gallic acid was quantified in the range of 7.09–72.14 μg/g, while vanillic acid was quantified in the range of 70.49 to 118.08 μg/g in selected Australian native herbs. Furthermore, 3-*p*-coumaroylquinic acid and 5-feruloylquinic acid were quantified in sea parsley (9.24 ± 0.08 μg/g) and bush mint (80.20 ± 5.11 μg/g), respectively, while 3-sinapoylquinic acid was quantified in bush tomatoes (102.26 ± 5.06 μg/g), bush mint (107.34 ± 4.98 μg/g), river mint (114.73 ± 2.41 μg/g), and sea parsley (89.28 ± 3.04 μg/g), respectively. Caffeic acid and protocatechuic acid were also quantified in all selected Australian native herbs and fruits (Table S2). The highest amount of caffeic acid was found in river mint (556.80 ± 29.28 μg/g), while the least concentration was measured in bush tomatoes (11.66 ± 0.58 μg/g). To the best of our knowledge, a very limited number of studies was conducted on Australian native bush mint, bush tomatoes, river mint, and sea parsley.

#### 2.6.2. Flavonoids

Flavonoids are the most abundant class of polyphenols widely present in fruits and vegetables [51]. A total of nine flavonoids were quantified in Australian native herbs and fruits (Table S2). Diosmetin (65.61 ± 2.04 μg/g), epicatechin gallate (67.79 ± 3.33 μg/g), acacetin (138.37 ± 22.49 μg/g), and luteolin (98.36 ± 1.73 μg/g) were only quantified in bush mint. Procyanidin B2 was only quantified in river mint (37.97 ± 4.10 μg/g) and bush tomatoes (44.15 ± 2.87 μg/g). Diosmin was quantified in bush mint (45.17 ± 2.96 μg/g), river mint (14.04 ± 0.47 μg/g), bush tomatoes (71.46 ± 5.57 μg/g), and sea parsley (10.64 ± 0.46 μg/g). Epicatechin was quantified in bush mint (39.90 ± 1.26 μg/g) and bush tomatoes (38.03 ± 0.19 μg/g). Kaempferol and kaempferol 3-glucoside were quantified in bush mint (64.16 ± 5.03 μg/g and 309.74 ± 50.04 μg/g) and sea parsley (12.48 ± 0.58 μg/g and 2.59 ± 0.18 μg/g), respectively.

#### 2.6.3. Other Polyphenols

A total of 11 other polyphenols including stilbenes, lignans, coumarins, phenolic terpenes, and other polyphenols were quantified in Australian native herbs. Sagerinic acid and umbelliferone were only quantified in bush mint (690.71 ± 61.64 μg/g and 112.63 ± 24.94 μg/g), while rosmanol was only quantified in bush tomatoes (56.45 ± 4.88 μg/g). This is the first time that we have identified and quantified sagerinic acid in bush mint. Polydatin and resveratrol were quantified in bush mint (39.67 ± 1.12 μg/g and 116.03 ± 4.09 μg/g) and bush tomatoes (32.44 ± 1.87 μg/g and 135.25 ± 5.05 μg/g), respectively. Coumarin, carnosol, and carnosic were quantified in bush mint and river mint. Pyrogallol was quantified in river mint (10.56 ± 0.45 μg/g), bush tomatoes (17.93 ± 0.77 μg/g), and sea parsley (19.16 ± 0.47 μg/g), respectively.

#### 2.6.4. Heatmap and Hierarchical Clustering of Quantified Phenolic Metabolites

**Figure 3:**
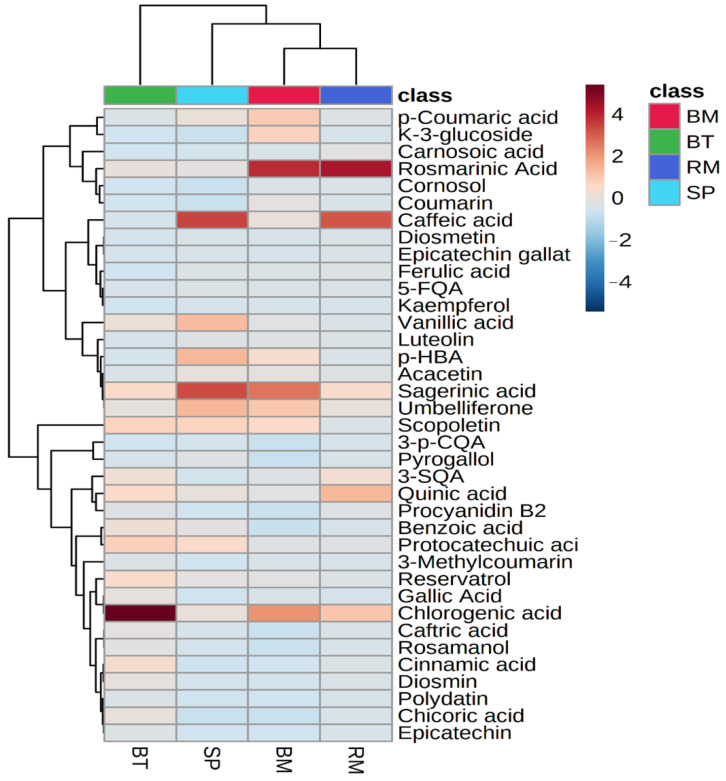
Heatmap clustering of quantified phenolic compounds in bush mint (BM), river mint (RM), bush tomatoes (BT), and sea parsley (SP).

A heatmap and hierarchical clustering were conducted to illustrate the concentration of phenolic metabolites quantified in Australian native herbs (Figure 3).

The variation of color indicates the concentration of phenolic metabolites. The blue color indicates the lower or zero concentration, while the red color indicates the higher concentration of phenolic metabolites. A total of three column-wise and sixteen row-wise clusters were generated, where bush mint and river mint were correlated to each other, while bush tomatoes were correlated to bush mint, river mint, and sea parsley. The highest concentration of rosmarinic acid was observed in bush mint and river mint, while chlorogenic acid was quantified with the highest concentration in bush tomatoes.

### 2.7. Pharmacokinetics Properties of the Abundant Phenolic Metabolites

The use of computational tools in drug discovery has increased. These methods are used to test the suitability of drug compounds for absorption, distribution, metabolism, excretion, and toxicology (ADMET) properties. Therefore, the study of drug compounds for ADMET properties is critical to improve the success rate of compounds during in vivo and clinical trials. In this context, we evaluated the ADMET properties of the most abundant phenolic compounds to improve the pharmaceutical potential of the selected native Australian medicinal plants in industrial and human utilization.

#### 2.7.1. Predicted Absorption and Distribution of Phenolic Compounds

**Figure 4:**
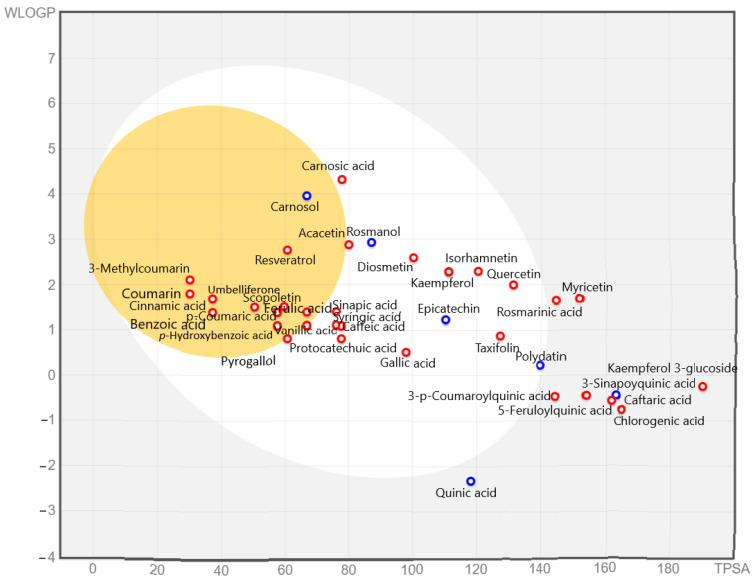
Boiled-Egg method for the evaluation of absorption of abundant phenolic compounds. The blue dots indicate molecules predicted to be effluated from the CNS by P-glycoprotein, and the red dots indicate molecules predicted not to be effluated from the CNS by P-glycoprotein. The egg yolk area predicts the phenolic compounds that passively penetrate the blood–brain barrier (BBB). The egg white area predicts which phenolic compounds have been absorbed through the gastrointestinal tract.

The absorption and distribution of the most phenolic species were predicted by the Boiled-Egg method and by following the previously reported protocol [15]. The obtained data are reported in Figure 4 and Tables S3 and S4.

The data in Figure 4 and Table S4 predicted that carnosol, coumarin, cinnamic acid, *p*-hydroxybenzoic acid, *p*-coumaric acid, 3-methylcoumarin, benzoic acid, resveratrol, umbelliferone, scopoletin, and ferulic acid readily passed through the blood–brain barrier (BBB), while protocatechuic acid, gallic acid, rosmanol, kaempferol, caffeic acid, acacetin, pyrogallol, carnosic acid, taxifolin, sinapic acid, vanillic acid, syringic acid, epicatechin, polydatin, quercetin, isorhamnetin, and diosmetin were absorbed through the gastrointestinal tract. In contrast, rosmarinic acid, quinic acid, caftaric acid, kaempferol 3-glucoside, myricetin, chlorogenic acid, 3-sinapoylquinic acid, 5-feroylquinic acid, and 3-*p*-coumaroylquinic acid did not predict gastrointestinal absorption.

Moreover, benzoic acid (100%), carnosic acid (99.03%), coumarin (97.34%), 3-methylcoumarin (97.26), scopoletin (95.28%), cinnamic acid (94.83%), umbelliferone (94.55%), acacetin (94.32%), ferulic acid (93.69%), *p*-coumaric acid (93.49%), rosmanol (93.41%), sinapic acid (93.06%), carnosol (91.21%), resveratrol (90.94%), *p*-hydroxybenzoic acid (83.96%), pyrogallol (83.55%), and luteolin (81.13%) were predicted with the highest human intestinal absorption, respectively (Table S3). Coumarin and 3-methylcoumarin predicted the skin permeability. It is worth noting that quinic acid and derivatives were predicted to have no human intestinal absorption (Table S3). Furthermore, cinnamic acid (1.72), benzoic acid (1.71), coumarin (1.65), 3-methylcoumarin (1.65), umbelliferone (1.21), *p*-coumaric acid (1.21), scopoletin (1.18), resveratrol (1.17), *p*-hydroxybenzoic acid (1.15), acacetin (1.14), pyrogallol (1.12), rosmanol (1.02), and taxifolin (0.92) were predicted to have the highest Caco-2 cells’ permeability, respectively. A phenolic compound has a high Caco-2 cell permeability if the Caco2 permeability value is higher than 0.90. The high Caco-2 absorption was predicted in accordance with a previous study [52]. Furthermore, the compounds which had Caco-2 permeability, gastrointestinal absorption, a good bioavailability score, and obeyed Lipinski’s rule of five and did not have BBB, did not act as a P-gp substrate, and had less skin permeability were successful drug compounds [53].

Rosmarinic acid was the most abundant phenolic acid quantified in bush mint and river mint, did not violate Lipinski’s rule and was insoluble in water. It predicted a low gastrointestinal absorption (32.52%) and negligible Caco-2 cells’ absorption. Previously, in vitro and in vivo experiments reported that rosmarinic acid has approximately 1% gastrointestinal absorption [54,55]. It was also predicted that rosmarinic acid cannot cross the BBB; see [54]. Caffeic acid predicted higher absorption (66.41%) than rosmarinic acid in accordance with the previously reported study [56]. Most of the phenolic compounds (around 95%) that are not absorbed in the gastrointestinal part can be metabolized by gut microbiota into small phenolic metabolites where they tend to absorb from the colon [57]. Generally, phenolic compounds are bound to albumin and transported to the liver through the portal vein after absorption [15]. On the other hand, the bioavailability of many phenolic compounds is low due to the limited absorption, extensive metabolism, and rapid excretion [58]. It is worth noting that the nature of phenolic compounds in the intestine can be altered due to the pre-systemic metabolism through sulphate conjugation, glucuronidation, and hydrogenation of the aliphatic double bonds [59].

#### 2.7.2. Drug Likeness

**Figure 5:**
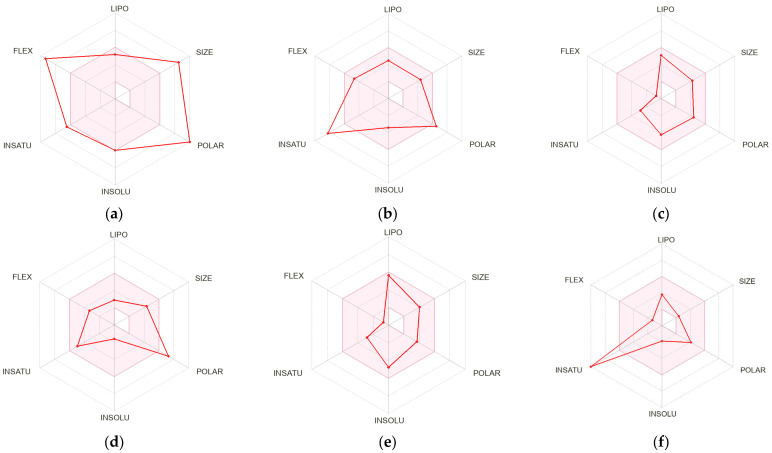
The pink area of the bioavailability radar represents the optimal range for each property. Bioavailability radars of sagerinic acid (**a**), rosmarinic acid (**b**), rosmanol (**c**), chlorogenic acid (**d**), carnosol (**e**), and caffeic acid (**f**) were obtained.

The oral bioavailability of phenolic compounds was predicted through the bioavailability radar using the method by Daina et al. [60]. The bioavailability radar was used to predict the drug likeness to assess the oral bioavailability of drug molecules (Figure 5).

Figure 5 and Table S5 depict that only carnosol and carnosic acid predicted the oral bioavailability. The oral bioavailability of the phenolic metabolites was predicted through the bioavailability radar by considering six parameters (size of compound, solubility, polarity, lipophilicity, saturation, and flexibility). Resveratrol was an important phenolic compound which predicted no oral bioavailability. The predicted results of resveratrol oral bioavailability are accordance with the previously published studies [61].

#### 2.7.3. Metabolism, Excretion, and Toxicity

It has been reported that cytochrome P450 (CYP) has a crucial role in the metabolism of phenolic and other drug molecules [58]. The predicted results of metabolism and excretion are reported in Table S6. The metabolism of phenolic drug compounds was predicted through the CYP (CYP3A4, CYP1A2, CYP2C9, CYP2C19, and CYP2D6) model for substrate or inhibitor. Phenolic metabolites that inhibited the CYP pathway may have triggered the accumulation and increased the concentration of phenolic compounds, which became the cause of higher toxicity of a particular compound and vice versa. Phenolic metabolites with higher total clearance were predicted with higher bioavailability and metabolism in the liver (Table S6). The overall bioavailability of resveratrol was low due to extensive and rapid metabolism and excretion [62].

The predicted results of the toxicological screening of individual phenolic metabolites are given in the Table S7. The predicted results indicate that all bioactive compounds did not inhibit the hERG 1 channel and most of the compounds did not predict skin sensitization, AIMES toxicity, *Tetrahymena pyriformis*, hepatotoxicity, and minnow toxicity, except a few compounds. Resveratrol (which is widely studied), stilbene, and 3-methylcoumarin predicted mutagenicity (AIMES toxicity), while resveratrol also predicted higher toxicity in *Tetrahymena pyriformis* at the rate of 0.29 μg/L. Previously, Patel et al. [62] also reported side effects of resveratrol in humans at the rate of 1 g/Kg body weight. Resveratrol, rosmanol, and 3-methylcoumarin predicted hepatotoxicity. Rosmanol also predicted minnow toxicity. Rosmarinic acid, chlorogenic acid, caffeic acid, and sagerinic acid were the most abundant phenolic compounds in the selected herbs which did not predict any toxicity. Previously, Hitl et al. [36] also reported no toxicity of rosmarinic acid in humans [63,64].

## 3. Materials and Methods

### 3.1. Chemicals and Reagents

Our previously published work describes all the chemicals used in this experiment [8,65,66]. All the pure standards were purchased from Sigma Aldrich (St. Louis, MI, USA).

### 3.2. Extraction Process of Phenolic Compounds

Bush mint, river mint, and sea parsley were purchased in dried form from Tucker Bush (https://tuckerbush.com.au) accessed on 21 September 2021. These plants were grown and dried in the Perth Hills in Whadjuk Noongar country. Bush tomatoes in dried form were purchased from Natif Australia (https://natif.com.au) accessed on 21 September 2021, and were further ground with a laboratory grinder. The phenolic compounds were extracted from these plants by taking 1 g sample in 20 mL 80% methanol acidified with 1% formic acid in triplicate. The complete process has been reported in our previously published work [65].

### 3.3. Polyphenols Estimation and Their Antioxidant Activities

#### 3.3.1. Quantification of TPC and TFC

The TPC and TFC of bush tomatoes, bush mint, river mint, and sea parsley were quantified by following the methods of Ali et al. [8] and Zahid et al. [67]. The standard curves of gallic acid (0–200 μg/mL) and quercetin (0–50 μg/mL) were generated to calculate the TPC and the TFC in this experiment.

#### 3.3.2. Antioxidant Activities

The DPPH and ABTS inhibition activities were quantified using the methods of Ali et al. [8] and Sharifi-Rad et al. [68]. The development of the calibration curve was completed by solutions of known 0–50 μg/mL ascorbic acid (C_6_H_8_O_6_) concentrations. Briefly, an aliquot of 25 μL of each extract or ascorbic acid was mixed with 275 μL 0.1 mM methanolic DPPH and placed in the dark for 25 min before reading the absorbance at 517 nm. The reducing properties of the culinary herbs were determined by following the method of Bashmil et al. [69]. An aliquot of 20 µL was mixed with 280 µL FRAP reagent (mixture of 20 mM ferric chloride, 10 mM TPTZ solution, and 300 mM sodium acetate buffer in the *v/v* ratio of 1:1:1). The mixture was kept for 10 min at 37 °C before the plate reading at 593 nm and results were expressed as mg AAE/g. An ABTS assay was conducted by modifying the method of Chou et al. [39]. The ABTS solution was prepared by dissolving 140 mM potassium persulfate and 7 mM ABTS solution both in water and placing them in the dark for 16 h. The next day, absorbance was set at 0.70 ± 0.02 by diluting with ethanol (approx. 1 mL in 45 mL of ethanol). The sample or standard (ascorbic acid) of 10 µL was mixed with a solution of 290 µL and incubated at room temperature in the dark for 6 min before reading the plate at 734 nm. Ascorbic acid (0–150 μg/mL) was used to generate a standard curve.

FICA was performed by a method of Ali et al. [8]. In total, 15 μL sample extract was mixed with 85 μL dist. water, 50 μL of 2 mM FeCl_3_ (with additional 1:15 dilution in water), and 50 μL of 5 mM ferrozine (with additional 1:6 dilution in water). Then, it was incubated at 25 °C for 10 min. Absorbance was measured at 562 nm and the equation was constructed by using 0–50 μg/mL EDTA. The PMA was measured by following the method of Sharifi-Rad et al. [68]. Briefly, 260 μL of phosphomolybdate reagent was mixed with 40 μL sample or standard. The phosphomolybdate reagent was prepared by mixing 0.004 M ammonium molybdate, 0.028 M sodium phosphate, and 0.6 M H_2_SO_4_ in an equal ratio. Then, the plate was incubated in a water bath at 95 ± 5 °C for 90 min by wrapping it with aluminum foil. After said time, the plate was cooled, and absorbance was measured at 695 nm. Ascorbic acid (0–200 μg/mL) was used to construct the equation. The method of Bashmil et al. [69], after modifications, was used to estimate RPA for these selected herbs. Briefly, the mixture was prepared as follows: 10 µL extract or standard, 25 µL of 0.2 M phosphate buffer (pH 6.6), and 25 µL of K_3_[Fe(CN)_6_] incubated for 20 min at 25 °C. Later, the addition of 25 µL 10% TCA solution in the mixture was followed by water and FeCl_3_, 85 µL and 10 µL, respectively. The mixture was again incubated at the same temperature for 20 min. Ascorbic acid (0–300 μg/mL) was used to establish the standard curve. The ^•^OH-RSA was determined by following the method of Ali et al. [8] with some modifications. The reaction mixture was prepared with 50 μL of each herbal extract, 6 mM FeSO_4_.7H_2_O, and 6 mM H_2_O_2_ (30%) and the mixture was incubated for 10 min at 25 °C before adding the 50 μL 6 mM 3-hydroxybenzoic acid. The standard curve was generated by using 0–300 μg/mL ascorbic acid at 510 nm, and the results were expressed as mg AAE/g of the sample.

### 3.4. LC-ESI-QTOF-MS/MS Analysis

The detailed identification and quantification of phenolic metabolites from bush tomatoes, bush mint, river mint, and sea parsley were conducted by following our previously established methods [65,66]. An Agilent 6520 Accurate Mass QTOF LC-MS (Agilent Technologies, Santa Clara, USA) in automatic MS/MS acquisition mode and a Synergi 4 um Hydro Reversed Phase (RP 80 Å) LC column (250 × 4.6 mm) connected with C18 ODS (4.0 × 2.0 mm) guard column were used in this experiment. An aliquot of 10 μL of each plant extract was injected, while the flow rate of mobile phase A (0.1% LC-MS grade formic acid in Milli-Q water) and mobile phase B (0.1% LC-MS grade formic acid in acetonitrile) was adjusted at 0.6 mL/min with the following gradient: 10–20% B (0–10 min), 20–25% B (10–20 min), 25–30% B (20–30 min), 30–45% B (30–40 min), 45–60% B (40–50 min), 60–90% B (50–65 min), 90–100% B (65–67 min), 100–10% B (67–68 min), and 10% B (68–70 min). The following LC conditions were followed: scan mode (50–1500 *m/z*), capillary voltage (3500 V), nebulization at 45 psi, nitrogen gas flow (9 L/min) at 325 °C, and collision energies (10, 20, 40 eV). Agilent MassHunter (version B.06.00) software was used for extraction and identification of individual phenolic compounds along with Personal Compound Database and Library (PCDL) library score 70, MassBank of North America (MoNA), and Human Metabolome Database (HMDB). Thirty-seven phenolic compounds were semi-quantified, while the MS/MS spectra of forty-one compounds were also acquired in this experiment.

### 3.5. Pharmacokinetics Study of the Most Abundant Phenolic Compounds

Pharmacokinetics properties were investigated by following the methods of Ali et al. [1,15]. Oral bioavailability, absorption, distribution, metabolism, excretion, and toxicity of the abundant phenolic compounds were predicted.

### 3.6. Statistical Analysis

XLSTAT-2019.1.3 (Addinsoft Inc. New York, NY, USA) software and Minitab version 18.0 (State College, PA, USA) were used for the investigation of the correlation analysis and analysis of variance in this study.

## 4. Conclusions

The results obtained from the given data indicate that bush mint has a strong antioxidant potential which is positively correlated to higher phenolic contents. A total of 123 phenolic metabolites were putatively identified in bush mint, bush tomatoes, river mint, and sea parsley. Phenolic metabolites from native Australian herbs and fruits may represent significant potential that could be used for additional health applications and against oxidative stress. Many of the phenolic compounds have been reported here for the first time in these native Australian herbs and medicinal plants. Rosmarinic acid, chlorogenic acid, sagerinic acid, caffeic acid, and quinic acid are the most abundant phenolic metabolites in these native Australian herbs. The phytochemical composition and strong antioxidant potential of these native Australian herbs and medicinal plants will explore the use of these plants in various food, feed, pharmaceutical, and cosmetic industries. Pharmacokinetic properties further help in the drug discovery of the identified compounds in these plants.

## Figures and Tables

**Table 1:** Pearson correlation between phenolic contents and antioxidant activities.

Variables	TPC	TFC	DPPH	ABTS	FRAP	PMA	^•^OH-RSA
TFC	0.96 **						
DPPH	0.84	0.76					
ABTS	0.45	0.46	0.80				
FRAP	0.96 **	0.85	0.91*	0.50			
PMA	0.76	0.84	0.82	0.84	0.68		
^•^OH-RSA	0.81	0.92 *	0.74	0.68	0.69	0.97 **	
FICA	0.99 **	0.91 *	0.83	0.38	0.98 **	0.67	0.72

** Significant correlation at *p* ≤ 0.05; * significant correlation at *p* ≤ 0.01.

**Table 2:** LC-MS/MS characterization of phenolic metabolites from Australian native herbs and fruits.

No.	RT (min)	Mode of Ionization	Theoretical (*m/z*)	Observed (*m/z*)	Mass Error (ppm)	MS/MS Productions	Molecular Formula	Proposed Compounds	Herbs and Fruits
								**Phenolic acids**	
								**Hydroxybenzoic acids**	
1	6.624	[M−H]^−^	331.0671	331.0682	3.3	169, 151, 125	C_13_H_16_O_10_	Gallic acid 4-*O*-glucoside	RM
2	7.054	** [M−H]^−^	169.0142	169.0134	−4.7	125	C_7_H_6_O_5_	* Gallic acid	BT, BM, SP
3	8.447	[M−H]^−^	315.0721	315.0715	−1.9	153, 109	C_13_H_16_O_9_	Protocatechuic acid 4-*O*-glucoside	SP, BM, BT
4	10.323	[M−H]^−^	167.0350	167.0350	0.0	152, 123, 108	C_8_H_8_O_4_	* Vanillic acid	BM, SP, BT
5	12.718	[M−H]^−^	153.0193	153.0193	0.0	109	C_7_H_6_O_4_	Protocatechuic acid	BM, SP, BT, RM
6	13.279	[M−H]^−^	299.0772	299.0788	5.3	255, 137	C_13_H_16_O_8_	4-Hydroxybenzoic acid 4-*O*-glucoside	BM, BT
7	16.152	[M−H]^−^	137.0244	137.0248	2.9	93, 65	C_7_H_6_O_3_	*p*-Hydroxybenzoic acid	SP, BM, BT
8	17.753	[M−H]^−^	121.0295	121.0295	0.0	103, 77	C_7_H_6_O_2_	* Benzoic acid	BT, RM, SP, BM
								**Hydroxycinnamic acids**	
9	4.122	[M−H]^−^	191.0561	191.0567	3.1	171, 127, 85	C_7_H_12_O_6_	Quinic acid	BT, RM, SP, BM
10	6.152	[M−H]^−^	311.0408	311.0411	0.9	267, 179, 135	C_13_H_12_O_9_	Caftaric acid	BT
11	7.342	** [M−H]^−^	355.0671	355.0673	0.6	179, 135	C_15_H_16_O_10_	Caffeic acid 3-*O*-glucuronide	BT, BM, SP, RM
12	12.816	[M−H]^−^	723.2142	723.2167	3.5	529, 499	C_33_H_40_O_18_	1-Sinapoyl-2-feruloylgentiobiose	BT, SP
13	13.212	[M−H]^−^	369.0827	369.0832	1.4	193, 178, 134	C_16_H_18_O_10_	Ferulic acid 4-*O*-glucuronide	BT, BM
14	14.722	** [M−H]^−^	325.0565	325.0571	1.8	193, 149	C_14_H_14_O_9_	Feruloyl tartaric acid	RM, BM
15	15.595	** [M−H]^−^	325.0929	325.0933	1.2	163, 119	C_15_H_18_O_8_	*p*-Coumaric acid 4-*O*-glucoside	BM, BT, RM, SP
16	16.282	[M−H]^−^	355.1034	355.1037	0.8	193, 176, 161, 134	C_16_H_20_O_9_	Ferulic acid 4-glucoside	BM, RM, BT
17	16.611	[M−H]^−^	223.0612	223.0608	−1.8	193, 179, 149, 134	C_11_H_12_O_5_	* Sinapic acid	BT
18	16.733	** [M−H]^−^	341.0878	341.0872	−1.8	179	C_15_H_18_O_9_	Caffeic acid 4-*O*-glucoside	BM, SP, BT
19	17.234	** [M−H]^−^	337.0929	337.0921	−2.4	191, 119	C_16_H_18_O_8_	3-*p*-Coumaroylquinic acid	BT, BM, SP
20	17.431	[M−H]^−^	385.1140	385.1144	1.0	223, 193	C_17_H_22_O_10_	1-*O*-Sinapoyl-ꞵ-d-glucose	RM, SP, BM, BT
21	17.558	** [M−H]^−^	295.0459	295.0473	4.7	115	C_13_H_12_O_8_	*p*-Coumaroyl tartaric acid	RM, SP, BM, BT
22	17.608	[M−H]^−^	163.0400	163.0395	−3.1	119	C_9_H_8_O_3_	* p-Coumaric acid	RM, BM, SP, BT
23	17.619	[M−H]^−^	193.0506	193.0504	−1.0	178, 149, 134	C_10_H_10_O_4_	* Ferulic acid	RM, SP
24	19.625	[M−H]^−^	367.1034	367.1036	0.5	191	C_17_H_20_O_9_	3-Feruloylquinic acid	BT, RM
25	19.701	[M−H]^−^	197.0450	197.0439	−5.7	182, 153, 138, 121	C_9_H_10_O_5_	* Syringic acid	BM, BT
26	21.648	[M−H]^−^	147.0451	147.0451	0.0	129, 103	C_9_H_8_O_2_	* Cinnamic acid	BM, RM, BT
27	23.958	[M−H]^−^	397.1140	397.1152	3.0	223, 191	C_18_H_22_O_10_	3-Sinapoylquinic acid	BT, RM
28	27.531	** [M−H]^−^	353.0878	353.0873	−1.4	191, 179, 161, 135	C_16_H_18_O_9_	* 3-Caffeoylquinic acid	SP, BT, BM, RM
29	27.531	** [M−H]^−^	515.1195	515.1196	0.2	191, 179, 135	C_25_H_24_O_12_	1,5-Dicaffeoylquinic acid	SP, BM
30	29.235	[M−H]^−^	179.0350	179.0353	1.7	135	C_9_H_8_O_4_	* Caffeic acid	BT, BM, SP, RM
31	29.423	[M−H]^−^	473.0725	473.0748	4.9	293, 311	C_22_H_18_O_12_	Chicoric acid	RM, BT
32	30.095	[M−H]^−^	959.2826	959.2826	0.0	887, 223, 207, 163	C_45_H_52_O_23_	1,2,2′-Trisinapoylgentiobiose	BT, BM
33	30.671	[M−H]^−^	359.0772	359.0770	−0.6	197, 179, 161, 135	C_18_H_16_O_8_	* Rosmarinic acid	BM, BT, SP, RM
34	32.125	[M−H]^−^	543.1508	543.1502	−1.1	193, 191, 134	C_27_H_28_O_12_	3,5-Diferuloylquinic acid	SP, BM, BT, RM
35	34.042	[M−H]^−^	693.2036	693.2037	0.1	193, 134	C_32_H_38_O_17_	1,2-Diferuloylgentiobiose	BM, SP, BT
								**Flavonoids**	
								**Flavanols**	
36	17.181	[M−H]^−^	577.1351	577.1353	0.3	451, 425, 407, 289	C_30_H_26_O_12_	* Procyanidin dimer B2	BT, RM
37	17.465	** [M−H]^−^	289.0717	289.0704	−4.5	245, 205, 179	C_15_H_14_O_6_	* Epicatechin	BT, BM
38	19.595	[M−H]^−^	865.1985	865.2004	2.2	739, 713, 695, 577, 451	C_45_H_38_O_18_	Procyanidin trimer C1	BM
39	19.625	** [M−H]^−^	481.0987	481.0999	2.5	305	C_21_H_22_O_13_	(-)-Epigallocatechin 3′-*O*-glucuronide	BT, SP, BM
40	21.869	[M−H]^−^	451.1246	451.1250	0.9	289, 245	C_21_H_24_O_11_	Catechin 3′-glucoside	BM, BT, SP
41	22.699	[M−H]^−^	1153.2619	1153.2599	−1.7	1135, 577, 289, 125	C_60_H_50_O_24_	Cinnamtannin A2	BM
42	25.918	[M−H]^−^	609.1250	609.1262	2.0	591, 539	C_30_H_26_O_14_	Prodelphinidin dimer B3	SP, RM, BT, BM
								**Flavanones**	
43	18.43	** [M−H]^−^	477.1038	477.1040	0.4	301	C_22_H_22_O_12_	Hesperetin 3′-*O*-glucuronide	SP, BM, BT
44	19.782	[M−H]^−^	595.1668	595.1665	−0.5	459, 287, 151	C_27_H_32_O_15_	Neoeriocitrin	BT, BM, RM
45	20.855	[M−H]^−^	433.1140	433.1139	−0.2	271	C_21_H_22_O_10_	Naringenin 7-*O*-glucoside	BT
46	22.011	[M−H]^−^	407.1864	407.1881	4.2	287, 243, 159, 119	C_25_H_28_O_5_	6-Geranylnaringenin	BT
47	24.293	[M−H]^−^	579.1719	579.1716	−0.5	459, 313, 271	C_27_H_32_O_14_	Naringin	RM, BT, SP, BM
48	39.991	[M−H]^−^	741.2247	741.2249	0.3	579	C_33_H_42_O_19_	Narirutin 4′-*O*-glucoside	BM, RM, BT, SP
49	52.783	[M−H]^−^	285.0768	285.0765	−1.0	243, 164, 151, 136	C_16_H_14_O_5_	Isosakuranetin	RM, BT
50	65.819	** [M+H]^+^	611.1971	611.1974	0.5	303	C_28_H_34_O_15_	Hesperidin	BM, BT, RM
								**Flavones**	
51	4.268	[M−H]^−^	637.1774	637.1754	−3.1	329	C_29_H_34_O_16_	Tricin 7-neohesperidoside	SP, BT
52	4.911	** [M−H]^−^	505.0987	505.1003	3.2	329	C_23_H_22_O_13_	Tricin 7-*O*-glucuronide	BM, RM
53	18.274	** [M−H]^−^	637.1046	637.1044	−0.3	285	C_27_H_26_O_18_	Luteolin 7-*O*-diglucuronide	BM, RM, BT
54	18.905	[M−H]^−^	285.0404	285.0418	4.8	151	C_15_H_10_O_6_	Kaempferol	BM, BT
55	19.447	** [M−H]^−^	343.0823	343.0814	−2.6	327, 255, 241	C_18_H_16_O_7_	Cirsilineol	BT, RM, BM
56	21.165	[M−H]^−^	577.1563	577.1555	−1.4	431, 269	C_27_H_30_O_14_	Rhoifolin	BT, BM, RM
57	25.466	[M−H]^−^	445.1140	445.1134	−1.3	325, 297, 282	C_22_H_22_O_10_	* Swertisin	BT, RM
58	25.83	** [M−H]^−^	285.0404	285.0403	−0.4	177, 151, 119	C_15_H_10_O_6_	3,4′,7-Tetrahydroxyflavone	BT, BM, SP, RM
59	27.21	** [M−H]^−^	593.1512	593.1513	0.2	449, 287	C_27_H_30_O_15_	Apigenin 6,8-di-C-glucoside	BT, BM, SP
60	27.29	** [M−H]^−^	607.1668	607.1668	0	300, 299	C_28_H_32_O_15_	* Diosmin	BT, RM, SP, BM
61	29.297	** [M−H]^−^	461.1089	461.1097	1.7	299	C_22_H_22_O_11_	Chrysoeriol 7-*O*-glucoside	BT, SP, RM
62	32.909	[M−H]^−^	431.0983	431.0993	2.3	269	C_21_H_20_O_10_	Apigenin 6-C-glucoside	BT, RM
63	41.946	[M−H]^−^	299.0561	299.0601	13.3	284	C_16_H_12_O_6_	* Diosmetin	RM
64	50.821	[M−H]^−^	343.0823	343.0809	−4.1	328, 313	C_18_H_16_O_7_	Santin	BM
65	52.842	[M−H]^−^	255.0658	255.0697	15.5	213, 171	C_15_H_12_O_4_	5,7-Dihydroxyflavanone	BM
66	53.416	[M−H]^−^	253.0506	253.0507	0.4	235, 151	C_15_H_10_O_4_	* Chrysin	BM
67	54.173	[M−H]^−^	283.0612	283.0642	10.7	268	C_16_H_12_O_5_	Wogonin	RM
68	68.447	[M+H]^+^	255.0652	255.0656	1.6	213, 137, 119	C_15_H_10_O_4_	7,4′-Dihydroxyflavone	SP
69	69.137	[M+H]^+^	303.0499	303.0493	−2.0	285, 169	C_15_H_10_O_7_	6-Hydroxyluteolin	RM
70	21.687	[M−H]^−^	283.0611	283.0639	9.9	268	C_16_H_12_O_5_	Acacetin	BT, BM
71	69.147	[M+H]^+^	317.0656	317.0652	−1.3	302	C_16_H_12_O_7_	8-Methoxyluteolin	BM
72	69.48	[M+H]^+^	549.1239	549.1258	3.5	531, 401, 301	C_25_H_24_O_14_	Chrysoeriol 7-*O*-(6″-malonyl-glucoside)	SP, BM, BT
								**Flavonols**	
73	6.656	[M−H]^−^	401.1242	401.1250	2.0	327, 209	C_21_H_22_O_8_	3-Methoxysinensetin	BT, RM
74	13.212	[M−H]^−^	623.1617	623.1627	1.6	315	C_28_H_32_O_16_	Isorhamnetin 3-*O*-rutinoside	BT, SP, BM, RM
75	13.358	[M−H]^−^	446.0854	446.0891	8.3	285	C_21_H_19_O_11_	Kaempferol 7-*O*-glucoside	RM
76	13.844	** [M−H]^−^	609.1097	609.1075	−3.6	301	C_26_H_26_O_17_	Quercetin 3-*O*-xylosyl-glucuronide	RM, BT, BM
77	14.873	** [M−H]^−^	609.1461	609.1459	−0.3	447, 285	C_27_H_30_O_16_	Kaempferol 3,7-*O*-diglucoside	BM, RM, SP, BT
78	19.099	** [M−H]^−^	625.1410	625.1425	2.4	317	C_27_H_30_O_17_	Myricetin 3-*O*-rutinoside	BT, SP
79	24.49	** [M−H]^−^	461.0725	461.0715	−2.2	285, 113, 85	C_21_H_18_O_12_	Kaempferol 3-*O*-glucuronide	BM, BT
80	25.494	[M−H]^−^	491.0831	491.0818	−2.6	315	C_22_H_20_O_13_	Isorhamnetin 3-*O*-glucuronide	BM
81	27.848	** [M−H]^−^	535.1093	535.1099	1.1	359	C_24_H_24_O_14_	Jaceidin 4′-*O*-glucuronide	BT, RM, BM
82	28.879	** [M−H]^−^	477.0674	477.0686	2.5	301	C_21_H_18_O_13_	Quercetin 4′-*O*-glucuronide	BT, RM, BM, SP
83	31.203	** [M−H]^−^	449.0725	449.0706	−4.2	317	C_20_H_18_O_12_	Myricetin 3-*O*-arabinoside	BM, SP, BT
84	31.977	** [M−H]^−^	269.0455	269.0453	−0.7	227, 151, 117	C_15_H_10_O_5_	Apigenin	BT, RM, BM
85	37.395	** [M−H]^−^	463.0882	463.0870	−2.6	317	C_21_H_20_O_12_	Myricetin 3-*O*-rhamnoside	BT, RM
86	44.404	** [M−H]^−^	329.0667	329.0679	3.6	314, 299, 271	C_17_H_14_O_7_	3,7-Dimethylquercetin	RM, BT, BM
								**Isoflavonoids**	
87	8.849	** [M−H]^−^	315.0874	315.0866	−2.5	300, 285, 135	C_17_H_16_O_6_	Violanone	RM, BM, SP, BT
88	12.208	[M−H]^−^	329.1030	329.1033	0.9	285, 163	C_18_H_18_O_6_	3′-*O*-Methylviolanone	SP, BT, BM
89	15.877	[M−H]^−^	457.1140	457.1131	−2.0	253	C_23_H_22_O_10_	6″-*O*-Acetyldaidzin	BM, RM
90	16.8	** [M−H]^−^	517.0987	517.1011	4.6	271	C_24_H_22_O_13_	6″-*O*-Malonylgenistin	BM, SP, RM, BT
91	20.242	[M−H]^−^	269.0819	269.0830	4.1	253, 239, 223	C_16_H_14_O_4_	Dihydroformononetin	BM, RM
92	21.949	[M−H]^−^	271.0976	271.0989	4.8	255, 149, 121	C_16_H_16_O_4_	3′-*O*-Methylequol	BM, BT
93	23.941	[M−H]^−^	591.1355	591.1357	0.3	415, 253	C_27_H_28_O_15_	Daidzin 4′-*O*-glucuronide	BM
94	28.073	** [M−H]^−^	531.1144	531.1175	5.8	283, 267	C_25_H_24_O_13_	6″-*O*-Malonylglycitin	BT
95	28.221	** [M−H]^−^	459.0933	459.0933	0.0	441, 283, 267	C_22_H_20_O_11_	Glycitein 4′-*O*-glucuronide	BT
96	28.286	[M−H]^−^	257.0819	257.0824	1.9	239, 135, 121	C_15_H_14_O_4_	3′,4′,7-Trihydroxyisoflavan	BT, BM
97	28.885	[M−H]^−^	487.1246	487.1262	3.3	283, 267, 59	C_24_H_24_O_11_	6″-*O*-Acetylglycitin	RM, BT
98	32.314	[M−H]^−^	299.0561	299.0558	−1.0	284	C_16_H_12_O_6_	3′-Hydroxymelanettin	BT, BM
99	53.600	[M−H]^−^	283.0612	283.0601	−3.8	268	C_16_H_12_O_5_	Biochanin A	BM
100	54.173	** [M−H]^−^	283.0612	283.0621	3.2	255	C_16_H_12_O_5_	2′-Hydroxyformononetin	RM, BM, SP
101	54.401	[M−H]^−^	285.0768	285.0767	−0.4	269, 203, 175	C_16_H_14_O_5_	Dihydrobiochanin A	BT, RM, BM, SP
102	56.229	[M−H]^−^	417.1191	417.1188	−0.7	241	C_21_H_22_O_9_	Equol 7-*O*-glucuronide	RM, BT, BM
								**Stilbenes**	
103	4.630	[M−H]^−^	243.0663	243.0643	−8.2	225, 201, 174, 159	C_14_H_12_O_4_	Piceatannol	SP, RM, BT
104	26.569	[M−H]^−^	419.1347	419.1348	0.2	257, 241	C_21_H_24_O_9_	Rhaponticin	BT
105	28.073	[M−H]^−^	227.0713	227.0709	−1.8	211, 167, 127	C_14_H_12_O_3_	* Resveratrol	BT
								**Lignans**	
106	26.010	[M−H]^−^	719.1612	719.1610	−0.3	360, 359, 197, 179, 161	C_36_H_32_O_16_	Sagerinic acid	BM
107	29.042	[M−H]^−^	357.1343	357.1348	1.4	341, 327, 191, 151	C_20_H_22_O_6_	Pinoresinol	RM, BT, SP
108	39.577	[M−H]^−^	557.2392	557.2392	0.0	539, 521, 509, 361	C_30_H_38_O_10_	Secoisolariciresinol-sesquilignan	SP, RM
109	47.844	[M−H]^−^	361.1656	361.1661	1.4	346, 177, 165	C_20_H_26_O_6_	Secoisolariciresinol	RM
110	49.423	[M−H]^−^	313.1081	313.1088	2.2	255	C_18_H_18_O_5_	2-Hydroxyenterolactone	BT, RM
111	56.027	[M−H]^−^	265.1234	265.1244	3.9	97	C_12_H_26_O_4_S	Magnolol	BM
112	69.400	** [M+H]^+^	299.1278	299.1279	0.3	281, 187, 165	C_18_H_18_O_4_	Enterolactone	RM, BM, SP
								**Other compounds**	
113	4.333	[M−H]^−^	191.0350	191.0355	2.6	175, 147	C_10_H_8_O_4_	Scopoletin	BM, SP, BT, RM
114	12.917	[M−H]^−^	339.0721	339.0731	2.9	177	C_15_H_16_O_9_	Aesculin	BT, BM, RM
115	18.146	[M−H]^−^	177.0193	177.0192	−0.6	133, 105	C_9_H_6_O_4_	Aesculetin	BT, BM, RM
116	19.445	[M−H]^−^	159.0451	159.0450	−0.6	115	C_10_H_8_O_2_	3-Methylcoumarin	BM
117	30.753	[M−H]^−^	161.0244	161.0242	−1.2	133	C_9_H_6_O_3_	Umbelliferone	BT, BM, SP, RM
118	37.435	[M−H]^−^	177.0557	177.0557	0.0	133	C_10_H_10_O_3_	Mellein	BT
119	39.116	** [M+H]^+^	147.0441	147.0441	0.0	103, 91	C_9_H_6_O_2_	Coumarin	RM, BM, BT
120	50.542	[M−H]^−^	345.1707	345.1714	2.0	301	C_20_H_26_O_5_	Rosmanol	BT
121	57.603	[M−H]^−^	329.1758	329.1770	3.6	285	C_20_H_26_O_4_	Carnosol	BM, RM
122	61.237	[M−H]^−^	331.1915	331.1910	−1.5	287	C_20_H_28_O_4_	Carnosic acid	RM, BM
123	6.624	[M−H]^−^	125.0244	125.0251	5.6	107, 97, 79	C_6_H_6_O_3_	* Pyrogallol	RM, BT, SP

RM = river mint; BM = bush mint; BT = bush tomatoes; SP = sea parsley; * = compounds were confirmed with pure standards; ** = compounds were identified in both modes.

## Data Availability

Not applicable.

## Supplementary Materials

The following supporting information can be downloaded at: https://www.mdpi.com/article/10.3390/plants12050993/s1, Figure S1: Total ion chromatograms (TIC) and base peak chromatograms (BPC) of bush mint, river mint, bush tomatoes, and sea parsley in positive (black color) and negative mode (blue color); Figure S2: MS/MS spectra of protocatechuic acid (A) and gallic acid (B); Figure S3: MS/MS spectra of caffeic acid, *p*-coumaric acid, and rosmarinic acid; Table S1: Phenolic contents in Australian native herbs and fruits and their antioxidant activities; Table S2: LC-MS/MS quantification of phenolic compounds (μg/g) from Australian native herbs and fruits; Table S3: Predicted absorption and distribution of selected compounds; Table S4: Pharmacokinetics properties of selected compounds; Table S5: Radar bioavailability properties of selected compounds; Table S6: Predicted metabolism and excretion of selected compounds; Table S7: Predicted toxicity of abundant phenolic compounds.
