# Supplementary material for: Characterization, Antioxidant Potential, and Pharmacokinetics Properties of Phenolic Compounds from Native Australian Herbs and Fruits
Source: Plants (Basel). 2023 Feb 21;12(5):993. doi: 10.3390/plants12050993 (PMC10005590; doi:10.3390/plants12050993)
Supplement: Supplementary file 1 [file plants-12-00993-s001.zip › plants-2250471-supplementary.pdf]

Supplementary Materials

Characterization, Antioxidant Potential, and Pharmacokinetics Study of Phenolic Compounds from Native Australian Plants

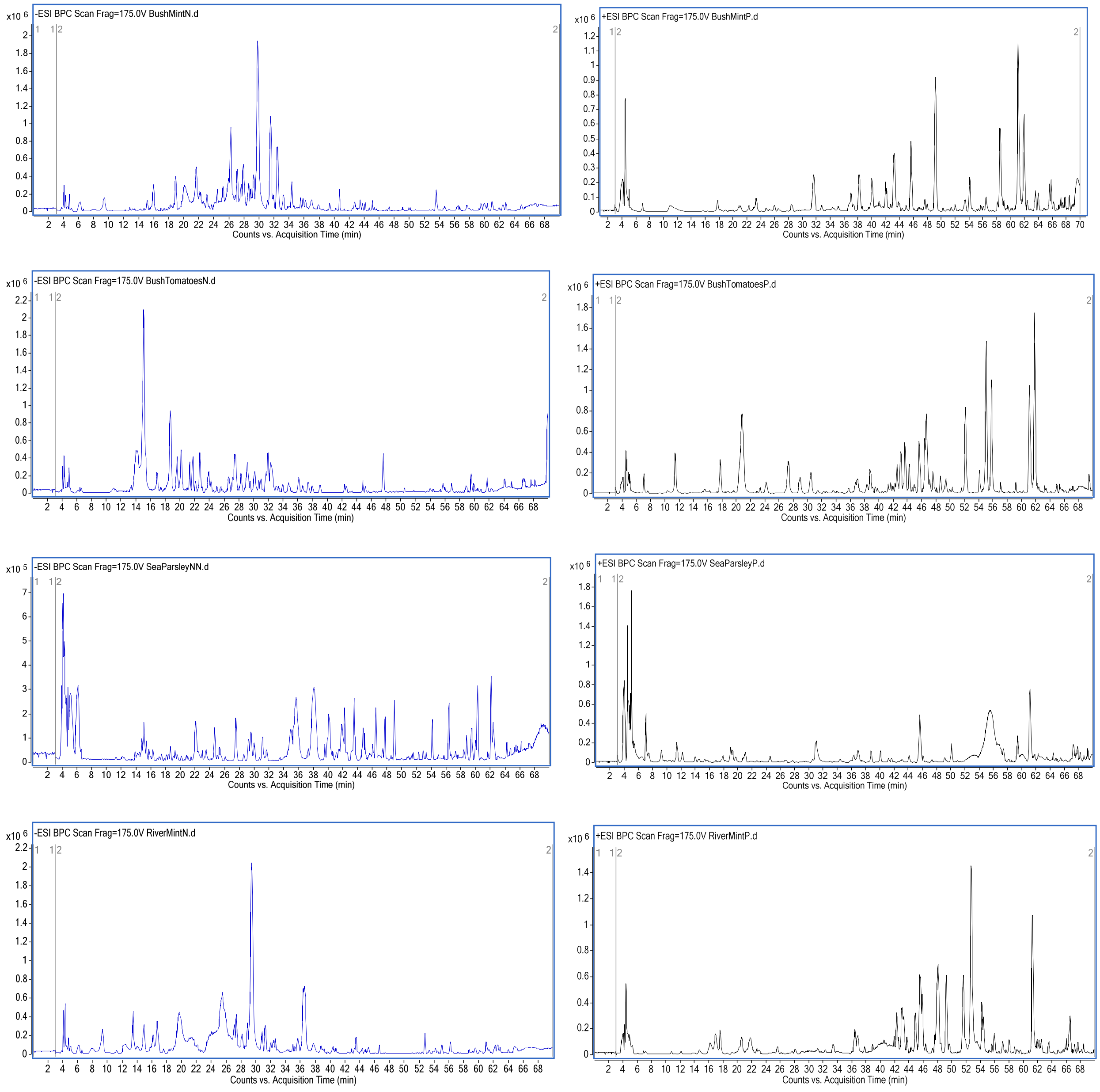

Figure S1. Base peak chromatograms (BPC) of bush mint, bush tomatoes, sea parsley, and river mint in positive (black color) and negative mode (blue color).

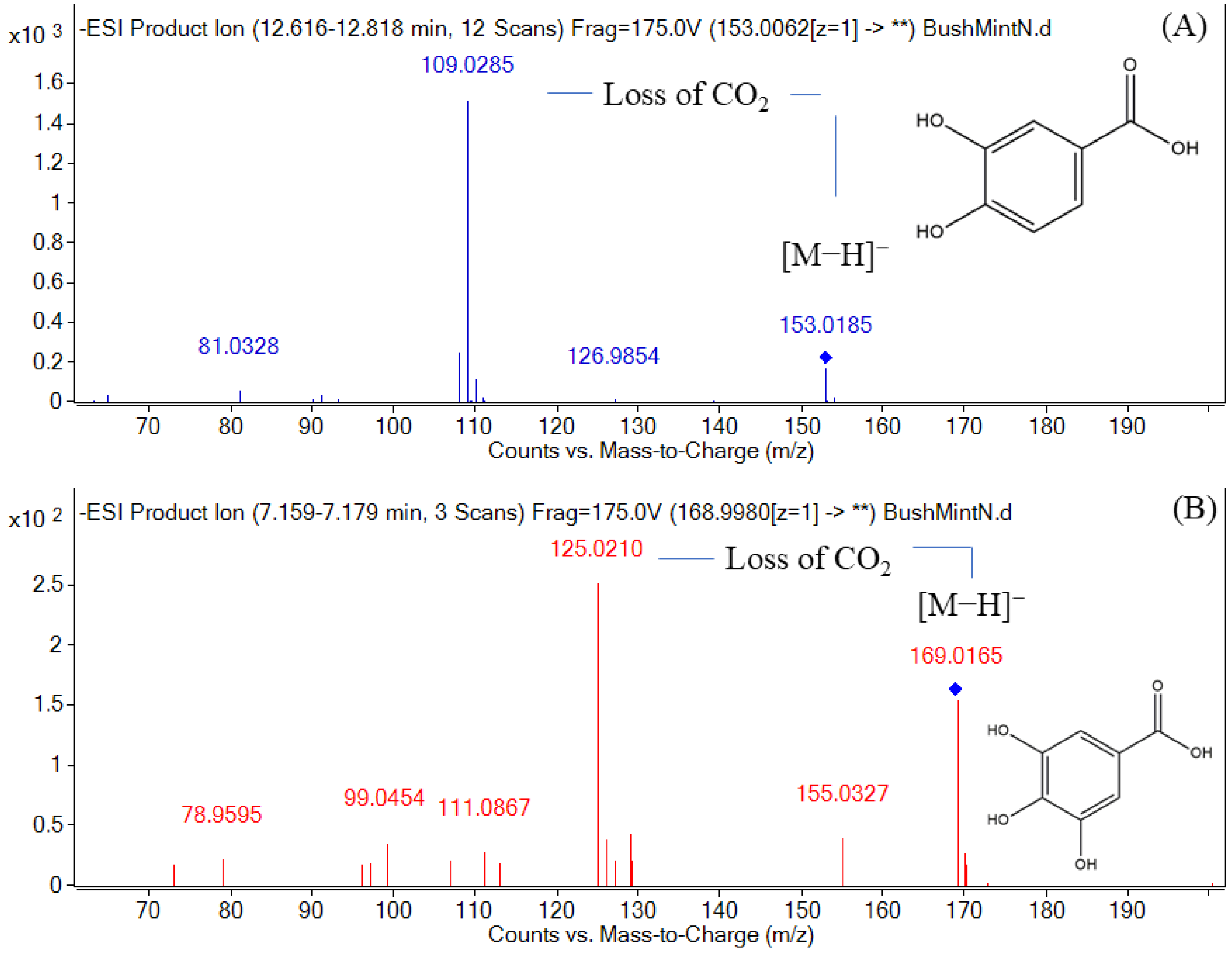

Figure S2. MS/MS spectra of protocatechuic acid (A) and gallic acid (B).

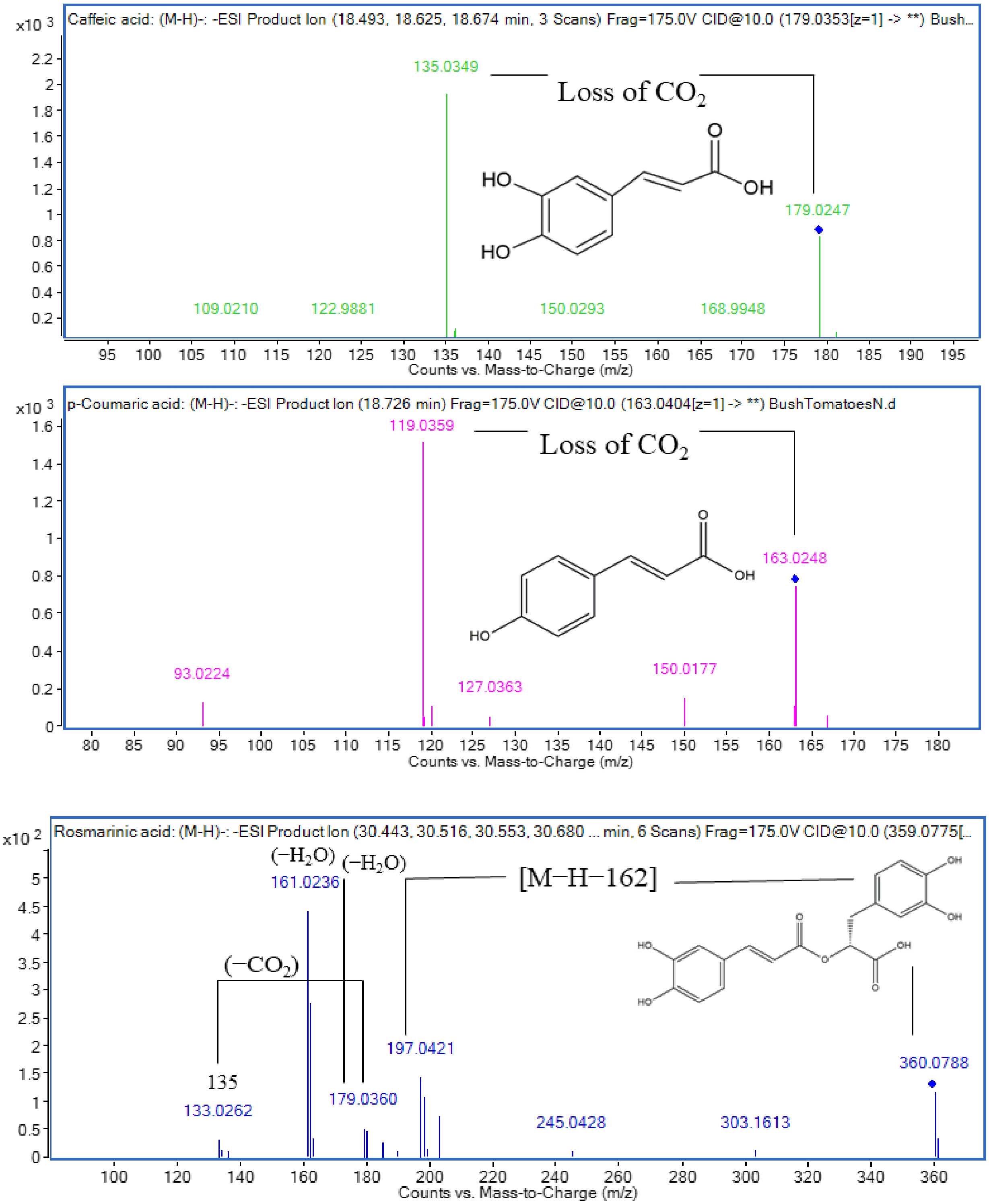

Figure S3. MS/MS spectra of caffeic acid, *p*-coumaric acid and rosmarinic acid.

Table S1. Phenolic contents in native Australian plants and fruits and their antioxidant activities

Variables	TPC (mg GAE/g)	TFC (mg QE/g)	DPPH (mg AAE/g)	ABTS (mg AAE/g)	FRAP (mg AAE/g)	PMA (mg AAE/g)	•OH-RSA (mg AAE/g)	FICA (mg EDTA/g)
River Mint	46.59 ± 1.34 [b]	13.73 ± 0.32 [b]	23.24 ± 1.23 [b]	66.85 ± 2.75 [b]	21.45 ± 2.88 [a]	13.41 ± 0.28 [c]	23.62 ± 0.47 [b]	3.02 ± 0.09 [a]
Bush Mint	57.70 ± 4.57 [a]	18.81 ± 1.14 [a]	28.86 ± 0.49 [a]	114.44 ± 1.01 [a]	23.02 ± 2.57 [a]	28.01 ± 0.95 [a]	43.25 ± 0.42 [a]	3.26 ± 0.10 [a]
Sea Parsley	13.44 ± 0.39 [d]	8.59 ± 0.51 [c]	12.47 ± 0.35 [c]	46.18 ± 0.38 [c]	5.13 ± 1.42 [c]	10.57 ± 0.18 [d]	19.66 ± 0.31 [c]	0.98 ± 0.10 [c]
Bush Tomato	26.78 ± 1.00 [c]	9.66 ± 0.42 [c]	24.85 ± 0.50 [b]	65.73 ± 2.38 [b]	15.13 ± 0.72 [b]	17.09 ± 0.38 [b]	23.32 ± 0.55 [b]	1.80 ± 0.10 [b]

TFC – total flavonoid content, TPC – total phenolic content; Values (n=3) are presented as mean ± standard deviation per gram powder. 2,2'-diphenyl-1-picrylhydrazyl (DPPH), 2,2'-azino-bis-3-ethylbenzenzothiazoline-6-sulfonic acid (ABTS), phosphomolybdate assay (PMA), reducing power assay (RPA), ferrous ion chelating assay (FICA), hydroxy-radical scavenging activity (•OH-RSA), ethylenediaminetetraacetic acid (EDTA), ferric reducing antioxidant power (FRAP). Values with superscript letters ([a-d]) are significantly different from each other.

Table S2. LC-MS/MS quantification of phenolic compounds (μg/g) from Australian native plants

No.	Compounds	BM	RM	BT	SP	Equation	R2
1	Rosmarinic Acid *	945.56 ± 43.50	745.67 ± 25.02	76.57 ± 4.98	23.43 ± 1.01	Y = 12943.4x − 76492	0.9999
2	3-*p*-Coumaroylquinic acid				9.24 ± 0.08	Y = 9657.4x − 40528	0.9999
3	3-Sinapoylquinic acid	107.34 ± 4.98	114.73 ± 2.41	102.26 ± 5.06	8.93 ± 0.30	Y = 9657.4x − 40528	0.9999
4	5-Feruloylquinic acid	80.20 ± 5.11				Y = 9657.4x − 40528	0.9999
5	*p*-Coumaric acid *	350.60 ± 8.59	30.96 ± 0.24	32.97 ± 0.23	30.63 ± 0.12	Y = 349225x + 507406	0.9932
6	*p*-Hydroxybenzoic acid *	224.80 ± 15.83			73.39 ± 15.95	Y = 63189x + 94570	0.9959
7	Gallic Acid *	70.48 ± 0.99		72.14 ± 1.50	7.09 ± 0.06	Y = 4E+06x − 127884	0.9959
8	Benzoic acid			95.31 ± 2.54	24.07 ± 2.27	Y = 4E+06x − 127884	0.9959
9	Caffeic acid *	167.43 ± 21.93	556.80 ± 29.28	11.66 ± 0.58	141.25 ± 5.11	Y = 6E+06x + 212752	0.9948
10	Caftaric acid			62.34 ± 0.91		Y = 9657.4x − 40528	0.9999
11	Chicoric acid		9.87 ± 0.41	74.03 ± 2.25		Y = 9657.4x − 40528	0.9999
12	Chlorogenic acid *	584.07 ± 12.39	238.76 ± 14.99	747.52 ± 67.48	29.07 ± 0.98	Y = 9657.4x − 40528	0.9999
13	Cinnamic acid *	36.87 ± 3.20	22.40 ± 1.68	125.13 ± 13.81		Y = 43521.7 − 32873	0.9941
14	Quinic acid	114.77 ± 12.51	284.52 ± 19.99	136.03 ± 13.73	28.22 ± 0.74	Y = 4E+06x − 127884	0.9959
15	Vanillic acid	118.19 ± 12.39		88.75 ± 2.53	70.49 ± 0.85	Y = 4E+06x − 127884	0.9959
16	Protocatechuic acid *	102.08 ± 15.47	35.47 ± 0.98	178.70 ± 19.34	43.57 ± 1.63	Y = 3E+06x − 48851	0.9998
17	Ferulic acid *	91.31 ± 2.56	30.77 ± 3.36		15.92 ± 0.38	Y = 91532x + 15195	0.9950
	Total phenolic acids	2993.7	2069.95	1803.41	505.3		
18	Diosmetin	65.61 ± 2.04				Y = 4E+06x − 72736	0.9987
19	Diosmin	45.17 ± 2.96	14.04 ± 0.47	71.46 ± 5.57	10.64 ± 0.46	Y = 4E+06x − 72736	0.9987
20	Epicatechin *	39.90 ± 1.26		38.03 ± 0.19		Y = 4E+06x − 72736	0.9987
21	Epicatechin gallate *	67.79 ± 3.33				Y = 3E+06x − 78678	0.9995
22	Kaempferol-3-glucoside *	309.74 ± 50.04			2.59 ± 0.18	Y = 2E+06x − 11882	0.9979
23	Kaempferol *	64.16 ± 5.03			12.48 ± 0.58	Y = 6E+06x − 19736	0.9997
24	Acacetin	138.37 ± 22.49				Y = 4E+06x − 72736	0.9987
25	Luteolin	98.36 ± 1.73				Y = 4E+06x − 72736	0.9987
26	Procyanidin B2		37.97 ± 4.10	44.15 ± 2.87		Y = 1E+06x − 38339	0.9989
	Total Flavonoids	829.1	52.01	153.64	25.71		
27	Polydatin *	39.67 ± 1.12		32.44 ± 1.87		Y = 535152x − 15101	0.9952
28	Resveratrol *	116.03 ± 4.09		135.25 ± 5.05		Y = 97707x − 476400	0.9974
29	Rosmanol			56.45 ± 4.88		Y = 535152x − 15101	0.9952
30	Sagerinic acid	690.71 ± 61.64				Y = 535152x − 15101	0.9952
31	Scopoletin	25.98 ± 2.51	13.22 ± 0.99	167.36 ± 21.07	51.55 ± 38.13	Y = 535152x − 15101	0.9952
32	Umbelliferone	112.63 ± 24.94				Y = 535152x − 15101	0.9952

33	3-Methylcoumarin	74.52 ± 1.25		36.68 ± 2.18		Y = 535152x − 15101	0.9952
34	Carnosic acid	72.29 ± 2.92	47.05 ± 3.27		9.54 ± 0.40	Y = 535152x − 15101	0.9952
35	Carnosol	82.25 ± 3.65	10.33 ± 0.33			Y = 535152x − 15101	0.9952
36	Coumarin	13.43 ± 0.97	10.49 ± 0.36			Y = 535152x − 15101	0.9952
37	Pyrogallol *		10.56 ± 0.45	17.93 ± 0.77	19.16 ± 0.47	Y = 53521.9 − 31891	0.9714
	Total other polyphenols	1227.51	91.65	446.11	80.25		

BM; Bush mint, RM; River mint, BT; Bush tomatoes, RM; River mint; compounds with asterisk (*) were used to generate equations.

Table S3. Predicted absorption and distribution of selected compounds.

		Absorption							Distribution			
No.	Compounds	Water solubility	Caco2 permeability	Intestinal absorption (human)	Skin Permeability	P-g substrate	P-g I inhibitor	P-g II inhibitor	VDss (human)	Fraction unbound (human)	BBB permeability	CNS permeability
1	3-*p*-Coumaroylquinic acid	-1.99	-0.66	43.93	-2.74	Yes	No	No	0.03	0.61	-1.16	-3.69
2	3-Sinapoylquinic acid	-2.55	-0.61	39.65	-2.74	Yes	No	No	-1.13	0.46	-1.40	-4.02
3	Caffeic acid	-2.33	0.63	69.41	-2.72	No	No	No	-1.10	0.53	-0.65	-2.61
4	Chlorogenic acid	-2.45	-0.84	36.38	-2.74	Yes	No	No	0.58	0.66	-1.41	-3.86
5	Cinnamic acid	-2.61	1.72	94.83	-2.70	No	No	No	-1.05	0.38	0.45	-1.83
6	Ferulic acid	-2.82	0.18	93.69	-2.72	No	No	No	-1.37	0.34	-0.24	-2.61
7	Gallic Acid	-2.56	-0.08	43.37	-2.74	No	No	No	-1.86	0.62	-1.10	-3.74
8	*p*-Coumaric acid	-2.38	1.21	93.49	-2.72	No	No	No	-1.15	0.43	-0.23	-2.42
9	*p*-Hydroxybenzoic acid	-1.88	1.15	83.96	-2.72	No	No	No	-1.56	0.59	-0.33	-3.21
10	Protocatechuic acid	-2.07	0.49	71.17	-2.73	No	No	No	-1.30	0.65	-0.68	-3.31
11	Quinic acid	-1.12	-0.26	32.27	-2.74	No	No	No	-0.22	0.82	-0.89	-3.67
12	Sinapic acid	-2.87	0.27	93.06	-2.73	Yes	No	No	-1.11	0.45	-0.25	-2.66
13	Syringic acid	-2.22	0.50	73.08	-2.74	Yes	No	No	-1.44	0.60	-0.19	-2.70
14	Rosmarinic acid	-3.06	-0.94	32.52	-2.74	Yes	No	No	0.39	0.35	-1.38	-3.35
15	Chicoric acid	-2.88	-0.96	8.17	-2.74	Yes	No	No	0.03	0.26	-1.81	-3.86
16	Caftaric acid	-2.54	-0.80	9.40	-2.74	No	No	No	-0.92	0.47	-1.23	-3.93
17	Benzoic acid	-1.74	1.71	100.00	-2.73	No	No	No	-1.64	0.52	-0.22	-2.00
18	Vanillic acid	-1.84	0.33	78.15	-2.73	No	No	No	-1.74	0.52	-0.38	-2.63
19	5-Feruloylquinic acid	-2.55	-0.56	44.76	-2.74	Yes	No	No	-1.30	0.39	-1.27	-3.94
20	Diosmin	-2.93	0.31	29.32	-2.74	Yes	No	No	1.43	0.11	-1.80	-4.84
21	Epicatechin	-3.12	-0.28	68.83	-2.74	Yes	No	No	1.03	0.24	-1.05	-3.30
22	Isorhamnetin	-3.00	0.00	76.01	-2.74	Yes	No	No	1.12	0.09	-1.14	-3.19
23	Kaempferol	-3.04	0.03	74.29	-2.74	Yes	No	No	1.27	0.18	-0.94	-2.23
24	Luteolin	-3.09	0.10	81.13	-2.74	Yes	No	No	1.15	0.17	-0.91	-2.25
25	Myricetin	-2.92	0.10	65.93	-2.74	Yes	No	No	1.32	0.24	-1.49	-3.71
26	Procyanidin B2	-2.89	-1.23	66.75	-2.74	Yes	Yes	Yes	-0.16	0.31	-1.94	-3.98
27	Quercetin	-2.93	-0.23	77.21	-2.74	Yes	No	No	1.56	0.21	-1.10	-3.07
28	Quercetin-3-glucoside	-2.93	0.24	48.00	-2.74	Yes	No	No	1.85	0.23	-1.69	-4.09
29	Taxifolin	-3.04	0.92	64.71	-2.74	Yes	No	No	1.64	0.32	-0.73	-3.20
30	Rutin	-2.89	-0.95	23.45	-2.74	Yes	No	No	1.66	0.19	-1.90	-5.18
31	Diosmetin	-3.24	0.33	79.90	-2.74	Yes	No	No	0.71	0.07	-0.95	-2.32

32	Kaempferol-3-glucoside	-2.86	0.31	48.05	-2.74	Yes	No	No	1.44	0.22	-1.51	-3.91
33	Acacetin	-3.28	1.14	94.32	-2.74	Yes	No	No	0.35	0.08	-0.20	-2.16
34	Coumarin	-1.52	1.65	97.34	-1.92	No	No	No	-0.14	0.37	-0.01	-1.93
35	Pyrogallol	-1.41	1.12	83.55	-2.75	No	No	No	0.13	0.71	-0.44	-3.25
36	Resveratrol	-3.18	1.17	90.94	-2.74	Yes	No	No	0.30	0.17	-0.05	-2.07
37	Umbelliferone	-2.13	1.21	94.55	-2.60	No	No	No	0.03	0.43	-0.28	-2.74
38	Carnosol	-4.12	0.57	91.21	-2.89	Yes	Yes	No	0.82	0.04	-0.10	-1.82
39	Carnosic acid	-3.22	0.80	99.03	-2.74	No	No	No	-1.03	0.05	-0.55	-2.00
40	Rosmanol	-3.61	1.02	93.41	-2.77	Yes	Yes	No	0.65	0.11	-0.58	-2.10
41	Sagerinic acid	-2.89	-1.69	2.09	-2.74	Yes	No	No	0.04	0.27	-2.56	-4.49
42	3-Methylcoumarin	-1.94	1.65	97.26	-1.81	Yes	No	No	-0.05	0.30	0.00	-1.89
43	Polydatin	-2.58	-0.08	51.09	-2.74	Yes	Yes	No	0.13	0.27	-1.03	-3.61
44	Scopoletin	-2.50	1.18	95.28	-2.94	No	No	No	0.03	0.36	-0.30	-2.32

Intestinal absorption (human) (IA), P-glycoprotein substrate, P-glycoprotein I inhibitor, P-glycoprotein II inhibitor, Water solubility (log mol/L) (WS), Volume of distributions (VDss); blood-brain barrier (BBB); central nervous system (CNS)

Interpretation of results: If the Caco2 permeability value is higher than 0.90, a compound has a high Caco-2 permeability. A compound with a value of less than 30% is poorly absorbed in the human intestinal. If a compound has a value less than −2.5 is considered to have a low skin permeability. The compounds with values < −3 cannot penetrate the central nervous system, while values > −2 are considered to penetrate the central nervous system. The compounds with values logBB < −1 are poorly distributed to the brain while values logBB > 0.3 are considered to cross the blood-brain barrier readily. VDss is considered low if below 0.71 L/kg (log VDss < −0.15) and high if above 2.81 L/kg (log VDss > 0.45).

Table S4. Pharmacokinetics properties of selected compounds

No.	Molecule	GI absorption	BBB permeant	P-gp substrate	CYP1A2 inhibitor	CYP2C19 inhibitor	CYP2C9 inhibitor	CYP2D6 inhibitor	CYP3A4 inhibitor	log Kp (cm/s) skin permeation
1	3-*p*-Coumaroylquinic acid	Low	No	No	No	No	No	No	No	-8.41
2	3-Sinapoylquinic acid	Low	No	Yes	No	No	No	No	No	-8.82
3	Caffeic acid	High	No	No	No	No	No	No	No	-6.58
4	Chlorogenic acid	Low	No	No	No	No	No	No	No	-8.76
5	Cinnamic acid	High	Yes	No	No	No	No	No	No	-5.69
6	Ferulic acid	High	Yes	No	No	No	No	No	No	-6.41
7	Gallic Acid	High	No	No	No	No	No	No	Yes	-6.84
8	*p*-Coumaric acid	High	Yes	No	No	No	No	No	No	-6.26
9	*p*-Hydroxybenzoic acid	High	Yes	No	No	No	No	No	No	-6.02
10	Protocatechuic acid	High	No	No	No	No	No	No	Yes	-6.42
11	Quinic acid	Low	No	Yes	No	No	No	No	No	-9.15
12	Sinapic acid	High	No	No	No	No	No	No	No	-6.63
13	Syringic acid	High	No	No	No	No	No	No	No	-6.77
14	Rosmarinic acid	Low	No	No	No	No	No	No	No	-6.82
15	Chicoric acid	Low	No	Yes	No	No	No	No	No	-7.77
16	Caftaric acid	Low	No	No	No	No	No	No	No	-8.16
17	Benzoic acid	High	Yes	No	No	No	No	No	No	-5.72
18	Vanillic acid	High	No	No	No	No	No	No	No	-6.31
19	5-Feruloylquinic acid	Low	No	No	No	No	No	No	No	-8.62
20	Diosmin	Low	No	Yes	No	No	No	No	No	-9.91
21	Epicatechin	High	No	Yes	No	No	No	No	No	-7.82
22	Isorhamnetin	High	No	No	Yes	No	No	Yes	Yes	-6.9
23	Kaempferol	High	No	No	Yes	No	No	Yes	Yes	-6.7
24	Luteolin	High	No	No	Yes	No	No	Yes	Yes	-6.25
25	Myricetin	Low	No	No	Yes	No	No	No	Yes	-7.4
26	Procyanidin B2	Low	No	No	No	No	No	No	Yes	-8.15
27	Quercetin	High	No	No	Yes	No	No	Yes	Yes	-7.05
28	Quercetin-3-glucoside	Low	No	No	No	No	No	No	No	-8.88
29	Taxifolin	High	No	No	No	No	No	No	No	-7.48
30	Rutin	Low	No	Yes	No	No	No	No	No	-10.26
31	Diosmetin	High	No	No	Yes	No	Yes	Yes	Yes	-5.93
32	Kaempferol-3-glucoside	Low	No	No	No	No	No	No	No	-8.52
33	Acacetin	High	No	No	Yes	No	Yes	Yes	Yes	-5.66
34	Coumarin	High	Yes	No	Yes	No	No	No	No	-6.2
35	Pyrogallol	High	Yes	No	No	No	No	No	Yes	-6.7
36	Resveratrol	High	Yes	No	Yes	No	Yes	No	Yes	-5.47

37	Umbelliferone	High	Yes	No	Yes	No	No	No	No	-6.17
38	Carnosol	High	Yes	Yes	No	No	Yes	No	No	-5.21
39	Carnosic acid	High	No	No	No	No	Yes	No	No	-4.86
40	Rosmanol	High	No	Yes	No	No	No	Yes	No	-5.99
41	Sagerinic acid	Low	No	No	No	No	No	No	No	-8.18
42	3-Methylcoumarin	High	Yes	No	Yes	No	No	No	No	-5.66
43	Polydatin	High	No	Yes	No	No	No	No	No	-7.95
44	Scopoletin	High	Yes	No	Yes	No	No	No	No	-6.39

Table S5. Radar bioavailability properties of selected compounds

No.	Compounds	MW	Rotatable bonds	XLOGP3	TPSA	WLOGP	ESOL Log S	Fraction Csp3	Lipinski violations	Bioavailability Score
1	3-p-Coumaroylquinic acid	338.31	5	-0.07	144.52	-0.46	-1.75	0.38	0	0.56
2	3-Sinapoylquinic acid	398.36	7	-0.13	162.98	-0.44	-1.92	0.44	0	0.11
3	Caffeic acid	180.16	2	1.15	77.76	1.09	-1.89	0.00	0	0.56
4	Chlorogenic acid	354.31	5	-0.42	164.75	-0.75	-1.62	0.38	1	0.11
5	Cinnamic acid	148.16	2	2.13	37.30	1.68	-2.37	0.00	0	0.85
6	Ferulic acid	194.18	3	1.51	66.76	1.39	-2.11	0.10	0	0.85
7	Gallic Acid	170.12	1	0.70	97.99	0.50	-1.64	0.00	0	0.56
8	p-Coumaric acid	164.16	2	1.46	57.53	1.38	-2.02	0.00	0	0.85
9	p-Hydroxybenzoic acid	138.12	1	1.58	57.53	1.09	-2.07	0.00	0	0.85
10	Protocatechuic acid	154.12	1	1.15	77.76	0.80	-1.86	0.00	0	0.56
11	Quinic acid	192.17	1	-2.37	118.22	-2.32	0.53	0.86	0	0.56
12	Sinapic acid	224.21	4	1.46	75.99	1.40	-2.16	0.18	0	0.56
13	Syringic acid	198.17	3	1.04	75.99	1.11	-1.84	0.22	0	0.56
14	Rosmarinic acid	360.31	7	2.36	144.52	1.65	-3.44	0.11	0	0.56
15	Chicoric acid	474.37	11	2.01	208.12	1.01	-3.58	0.09	2	0.11
16	Caftaric acid	312.23	7	0.06	161.59	-0.56	-1.55	0.15	0	0.11
17	Benzoic acid	122.12	1	1.87	37.30	1.38	-2.20	0.00	0	0.85
18	Vanillic acid	168.15	2	1.43	66.76	1.10	-2.02	0.12	0	0.85
19	5-Feruloylquinic acid	368.34	6	-0.10	153.75	-0.45	-1.84	0.41	0	0.11
20	Diosmin	608.54	7	0.14	238.20	-1.09	-3.51	0.46	3	0.17
21	Epicatechin	290.27	1	0.36	110.38	1.22	-2.22	0.20	0	0.55
22	Isorhamnetin	316.26	2	1.87	120.36	2.29	-3.36	0.06	0	0.55
23	Kaempferol	286.24	1	1.90	111.13	2.28	-3.31	0.00	0	0.55
24	Luteolin	286.24	1	2.53	111.13	2.28	-3.71	0.00	0	0.55
25	Myricetin	318.24	1	1.18	151.59	1.69	-3.01	0.00	1	0.55
26	Procyanidin B2	578.52	3	2.37	220.76	2.35	-5.14	0.20	3	0.17
27	Quercetin	302.24	1	1.54	131.36	1.99	-3.16	0.00	0	0.55
28	Quercetin-3-glucoside	464.38	4	0.36	210.51	-0.54	-3.04	0.29	2	0.17
29	Taxifolin	304.25	1	0.95	127.45	0.86	-2.66	0.13	0	0.55

30	Rutin	610.52	6	-0.33	269.43	-1.69	-3.30	0.44	3	0.17
31	Diosmetin	300.26	2	3.10	100.13	2.59	-4.06	0.06	0	0.55
32	Kaempferol-3-glucoside	448.38	4	0.72	190.28	-0.24	-3.18	0.29	2	0.17
33	Acacetin	284.26	2	3.35	79.90	2.88	-4.14	0.06	0	0.55
34	Coumarin	146.14	0	1.39	30.21	1.79	-2.29	0.00	0	0.55
35	Pyrogallol	126.11	0	0.52	60.69	0.80	-1.44	0.00	0	0.55
36	Resveratrol	228.24	2	3.13	60.69	2.76	-3.62	0.00	0	0.55
37	Umbelliferone	162.14	0	1.58	50.44	1.50	-2.46	0.00	0	0.55
38	Carnosol	330.42	1	4.38	66.76	3.96	-4.77	0.65	0	0.55
39	Carnosic acid	332.43	2	4.89	77.76	4.32	-5.03	0.65	0	0.56
40	Rosmanol	346.42	1	3.41	86.99	2.93	-4.25	0.65	0	0.55
41	Sagerinic acid	720.63	14	3.54	289.04	2.92	-5.96	0.22	3	0.11
42	3-Methylcoumarin	160.17	0	2.28	30.21	2.10	-2.89	0.10	0	0.55
43	Polydatin	390.38	5	1.03	139.84	0.23	-2.90	0.30	1	0.55
44	Scopoletin	192.17	1	1.53	59.67	1.51	-2.46	0.10	0	0.55

(Lipophilicity: XLOGP3 between −0.7 and +5.0, size: MW between 150 and 500 g/mol, polarity: TPSA between 20 and 130Å, solubility: log S not higher than 6, saturation: fraction of carbons in the sp3 hybridization not less than 0.25, and flexibility: no more than nine rotatable bonds; topological polar surface area (TPSA)

Table S6. Predicted metabolism and excretion of selected compounds.

		Metabolism							Excretion	
No.	Compounds	CYP2D6 substrate	CYP3A4 substrate	CYP1A2 inhibitor	CYP2C19 inhibitor	CYP2C9 inhibitor	CYP2D6 inhibitor	CYP3A4 inhibitor	Total Clearance	Renal OCT2 substrate
1	3-*p*-Coumaroylquinic acid	No	No	No	No	No	No	No	0.45	No
2	3-Sinapoylquinic acid	No	No	No	No	No	No	No	0.73	No
3	Caffeic acid	No	No	No	No	No	No	No	0.51	No
4	Chlorogenic acid	No	No	No	No	No	No	No	0.31	No
5	Cinnamic acid	No	No	No	No	No	No	No	0.78	No
6	Ferulic acid	No	No	No	No	No	No	No	0.62	No
7	Gallic Acid	No	No	No	No	No	No	No	0.52	No
8	*p*-Coumaric acid	No	No	No	No	No	No	No	0.66	No
9	*p*-Hydroxybenzoic acid	No	No	No	No	No	No	No	0.59	No
10	Protocatechuic acid	No	No	No	No	No	No	No	0.55	No
11	Quinic acid	No	No	No	No	No	No	No	0.64	No
12	Sinapic acid	No	No	No	No	No	No	No	0.72	No
13	Syringic acid	No	No	No	No	No	No	No	0.65	No
14	Rosmarinic acid	No	No	No	No	No	No	No	0.25	No
15	Chicoric acid	No	No	No	No	No	No	No	0.06	No
16	Caftaric acid	No	No	No	No	No	No	No	0.45	No
17	benzoic acid	No	No	No	No	No	No	No	0.71	No
18	Vanillic acid	No	No	No	No	No	No	No	0.63	No
19	5-Feruloylquinic acid	No	No	No	No	No	No	No	0.39	No
20	Diosmin	No	No	No	No	No	No	No	-0.11	No
21	Epicatechin	No	No	No	No	No	No	No	0.18	No
22	Isorhamnetin	No	No	Yes	No	No	No	No	0.51	No
23	Kaempferol	No	No	Yes	No	No	No	No	0.48	No
24	Luteolin	No	No	Yes	No	Yes	No	No	0.50	No
25	Myricetin	No	No	Yes	No	No	No	No	0.42	No
26	Procyanidin B2	No	No	No	No	No	No	No	-0.09	Yes
27	Quercetin	No	No	Yes	No	No	No	No	0.41	No

28	Quercetin-3-glucoside	No	No	No	No	No	No	No	0.39	No
29	Taxifolin	No	No	No	No	No	No	No	-0.08	No
30	Rutin	No	No	No	No	No	No	No	-0.37	No
31	Diosmetin	No	No	Yes	Yes	Yes	No	No	0.60	No
32	Kaempferol-3-glucoside	No	No	No	No	No	No	No	0.46	No
33	Acacetin	No	Yes	Yes	Yes	Yes	No	Yes	0.66	No
34	Coumarin	No	No	Yes	No	No	No	No	0.97	No
35	Pyrogallol	No	No	No	No	No	No	No	0.10	No
36	Resveratrol	No	Yes	Yes	Yes	No	No	No	0.08	No
37	Umbelliferone	No	No	Yes	No	No	No	No	0.71	No
38	Carnosol	No	Yes	No	Yes	No	No	No	0.28	No
39	Carnosic acid	No	No	No	No	No	No	No	0.38	No
40	Rosmanol	No	No	No	No	No	No	No	0.29	No
41	Sagerinic acid	No	No	No	No	No	No	No	-0.92	No
42	3-Methylcoumarin	No	No	Yes	No	No	No	No	0.96	No
43	Polydatin	No	No	No	No	No	No	No	0.06	No
44	Scopoletin	No	No	Yes	No	No	No	No	0.73	No

Organic cation transporter 2 (OCT2)

Table S7. Predicted toxicity of abundant phenolic compounds

No.	Compounds	AMES toxicity	MTD-H	hERG I inhibitor	hERG II inhibitor	ORAT (LD50)	LOAEL	Hepatotoxicity	Skin Sensitization	*T. pyriformis* toxicity	Minnow toxicity
1	3-*p*-Coumaroylquinic acid	No	-0.09	No	No	1.74	2.51	No	No	0.29	4.61
2	3-Sinapoylquinic acid	No	0.86	No	No	1.99	3.81	No	No	0.29	5.24
3	Caffeic acid	No	1.15	No	No	2.38	2.09	No	No	0.29	2.25
4	Chlorogenic acid	No	-0.13	No	No	1.97	2.98	No	No	0.29	5.74
5	Cinnamic acid	No	1.11	No	No	2.09	2.65	No	No	0.25	1.72
6	Ferulic acid	No	1.08	No	No	2.28	2.07	No	No	0.27	1.83
7	Gallic Acid	No	0.70	No	No	2.22	3.06	No	No	0.29	3.19
8	*p*-Coumaric acid	No	1.11	No	No	2.16	2.53	No	No	0.32	1.61
9	*p*-Hydroxybenzoic acid	No	0.85	No	No	2.26	2.48	No	No	0.27	1.81
10	Protocatechuic acid	No	0.81	No	No	2.42	2.02	No	No	0.27	2.45
11	Quinic acid	No	1.63	No	No	1.13	3.53	No	No	0.29	4.87
12	Sinapic acid	No	1.19	No	No	2.24	2.32	No	No	0.26	2.18
13	Syringic acid	No	1.37	No	No	2.16	2.42	No	No	0.28	2.55
14	Rosmarinic acid	No	0.15	No	No	2.81	2.91	No	No	0.30	2.70
15	Chicoric acid	No	0.23	No	No	2.45	4.06	No	No	0.29	4.19
16	Caftaric acid	No	0.93	No	No	2.17	4.23	No	No	0.29	4.87
17	benzoic acid	No	0.61	No	No	2.17	2.64	No	No	0.09	1.84
18	Vanillic acid	No	0.72	No	No	2.45	2.03	No	No	0.27	1.93
19	5-Feruloylquinic acid	No	1.29	No	No	2.03	4.49	No	No	0.29	4.88
20	Diosmin	No	0.57	No	Yes	2.51	3.34	No	No	0.29	5.35
21	Epicatechin	No	0.44	No	No	2.43	2.50	No	No	0.35	3.59
22	Isorhamnetin	No	0.58	No	No	2.41	2.50	No	No	0.30	2.21
23	Kaempferol	No	0.53	No	No	2.45	2.51	No	No	0.31	2.89
24	Luteolin	No	0.50	No	No	2.46	2.41	No	No	0.33	3.17
25	Myricetin	No	0.51	No	No	2.50	2.72	No	No	0.29	5.02
26	Procyanidin B2	No	0.44	No	Yes	2.48	4.35	No	No	0.29	8.70
27	Quercetin	No	0.50	No	No	2.47	2.61	No	No	0.29	3.72
28	Quercetin-3-glucoside	No	0.57	No	Yes	2.54	4.42	No	No	0.29	8.06
29	Taxifolin	No	0.35	No	No	2.26	3.10	No	No	0.29	4.69
30	Rutin	No	0.45	No	Yes	2.49	3.67	No	No	0.29	7.68
31	Diosmetin	No	0.42	No	No	2.34	2.27	No	No	0.34	1.74

32	Kaempferol-3-glucoside	No	0.58	No	No	2.55	4.53	No	No	0.29	6.74
33	Acacetin	No	0.09	No	No	2.22	1.26	No	No	0.42	1.00
34	Coumarin	No	0.44	No	No	2.11	1.90	No	No	0.37	1.56
35	Pyrogallol	No	-0.27	No	No	2.05	2.37	No	No	0.13	2.73
36	Resveratrol	Yes	0.33	No	No	2.53	1.53	No	No	0.75	1.52
37	Umbelliferone	No	0.69	No	No	2.05	1.75	Yes	No	0.55	1.71
38	Carnosol	No	0.23	No	No	2.19	1.91	No	No	0.41	-0.64
39	Carnosic acid	No	0.35	No	No	2.89	1.97	No	No	0.29	-0.63
40	Rosmanol	No	0.33	No	Yes	1.98	2.55	Yes	No	0.33	0.29
41	Sagerinic acid	No	0.44	No	No	2.48	5.63	No	No	0.29	6.47
42	3-Methylcoumarin	Yes	0.27	No	No	2.07	1.90	Yes	No	0.55	0.84
43	Polydatin	No	0.57	No	No	2.52	4.47	No	No	0.29	4.39
44	Scopoletin	No	0.61	No	No	1.95	1.38	No	No	0.52	1.61

Oral Rat Acute Toxicity (ORAT), Oral Rat Chronic Toxicity (LOAEL), Max. tolerated dose (human) (MTD-H)

Explanations

Minnow toxicity

LC50 values below 0.5mM (log LC50 < −0.3) are regarded as high acute toxicity.

T. Pyriformis toxicity

pIGC50 (negative logarithm of the concentration required to inhibit 50% growth in log µg/L) is considered, with a value > −0.5 log µg/L is considered toxic.

AMES toxicity: if the AMES value is positive, the compound will be mutagenic.

Maximum tolerated dose: a value less than is equal to 0.477 log(mg/kg/day) is considered low and high if greater than 0.477 log(mg/kg/day).
